# The full-length structure of *Thermus scotoductus* OLD defines the ATP hydrolysis properties and catalytic mechanism of Class 1 OLD family nucleases

**DOI:** 10.1093/nar/gkaa059

**Published:** 2020-02-03

**Authors:** Carl J Schiltz, Myfanwy C Adams, Joshua S Chappie

**Affiliations:** Department of Molecular Medicine, Cornell University, Ithaca, NY 14853, USA

## Abstract

OLD family nucleases contain an N-terminal ATPase domain and a C-terminal Toprim domain. Homologs segregate into two classes based on primary sequence length and the presence/absence of a unique UvrD/PcrA/Rep-like helicase gene immediately downstream in the genome. Although we previously defined the catalytic machinery controlling Class 2 nuclease cleavage, degenerate conservation of the C-termini between classes precludes pinpointing the analogous residues in Class 1 enzymes by sequence alignment alone. Our Class 2 structures also provide no information on ATPase domain architecture and ATP hydrolysis. Here we present the full-length structure of the Class 1 OLD nuclease from *Thermus scotoductus* (Ts) at 2.20 Å resolution, which reveals a dimerization domain inserted into an N-terminal ABC ATPase fold and a C-terminal Toprim domain. Structural homology with genome maintenance proteins identifies conserved residues responsible for Ts OLD ATPase activity. Ts OLD lacks the C-terminal helical domain present in Class 2 OLD homologs yet preserves the spatial organization of the nuclease active site, arguing that OLD proteins use a conserved catalytic mechanism for DNA cleavage. We also demonstrate that mutants perturbing ATP hydrolysis or DNA cleavage *in vitro* impair P2 OLD-mediated killing of *recBC*^−^*Escherichia coli* hosts, indicating that both the ATPase and nuclease activities are required for OLD function *in vivo*.

## INTRODUCTION

Overcoming lysogenization defect (OLD) proteins comprise a poorly characterized family of nucleases that contain an N-terminal ATPase domain and C-terminal **Top**isomerase/**Prim**ase (Toprim) catalytic domain (1,2). OLD homologs are widely conserved in bacteria, archaea and some viruses and can be subdivided into two classes (3). Class 1 OLD homologs exist as single, isolated genes while Class 2 *old* genes always appear in tandem with a UvrD/PcrA/Rep-like helicase. The coding sequences of Class 1 proteins are also on average ∼50 amino acids shorter than their Class 2 counterparts. Despite widespread prevalence across a plethora of species, little is known about the exact biological function of these enzymes. The Class 1 OLD homolog from the temperate bacteriophage P2 remains the best characterized to date. Genetic studies showed *old^+^* P2 lysogens kill *Escherichia coli recB* and *recC* mutant hosts after infection and specifically interfere with bacteriophage λ growth (4,5). Further *in vitro* characterization of recombinant P2 OLD revealed DNA exonuclease activity and ribonuclease activity (6). A saturating genome-wide transposon screen of *Salmonella typhimurium* indicated that the *old* gene is conditionally essential in some instances like temperature stress (7), but the underlying mechanism for this phenotypic observation has yet to be clarified.

We previously demonstrated that the Class 2 OLD homologs from *Burkholderia pseudomallei* and *Xanthomonas campestris* pv. *campestris* function as metal-dependent nucleases *in vitro* and described the crystal structures of their catalytic C-terminal regions (CTRs) (3). Class 2 CTRs contain a Toprim domain with altered architecture and a unique helical domain. Side chains in both domains contribute to the nuclease active site and adopt a geometry that supports a two-metal catalysis mechanism for cleavage. Degenerate sequence conservation between the C-termini of Class 1 and Class 2 homologs, however, precludes pinpointing the analogous side chains in Class 1 enzymes by alignment alone. Thus, it remains unclear whether Class 1 enzymes like that from P2 utilize the same mechanism and cleavage machinery. Moreover, these Class 2 CTR models provide no information on the architecture of the ATPase domain or the ATP hydrolysis machinery, as the N-terminal region (NTR) common to both classes was removed for crystallization purposes. This constraint has hindered our ability to understand nuclease function and regulation in the context of a full-length protein.

Here, we present the crystal structure of a full-length Class 1 OLD nuclease from *Thermus scotoductus* (Ts) at 2.20 Å resolution. The structure reveals a three domain architecture with a dimerization domain inserted into the N-terminal ABC ATPase domain and a C-terminal Toprim domain. The ATPase domains share structural homology with genome maintenance proteins, which identifies the critical side chains responsible for Ts OLD ATP hydrolysis and highlights sequence variations that are unique to both classes of OLD proteins. The orientation of the ATPase domains within the apo Ts OLD dimer differs significantly from the nucleotide-bond conformations of other DNA repair ABC ATPases, suggesting additional nucleotide-dependent conformational rearrangements may occur. Surprisingly, the Ts OLD C-terminus lacks the helical domain present in Class 2 OLD homologs yet preserves the spatial organization of the nuclease active site, arguing that OLD proteins use a conserved catalytic mechanism for DNA cleavage. Moreover, we show that mutants perturbing ATP hydrolysis or DNA cleavage *in vitro* abolish P2 OLD-mediated killing of *recBC* deficient *E. coli* hosts, indicating that both the ATPase and nuclease activities are required for OLD function *in vivo*. Together our data provide novel insights into the structure and mechanisms of Class 1 OLD proteins and further define the basic biological properties of the OLD family as a whole.

## MATERIALS AND METHODS

### Cloning, expression and purification of Ts OLD

DNA encoding *T. scotoductus* OLD (UniProt E8PLM2) was codon optimized for *E. coli* expression and synthesized commercially by IDT. Full-length (FL, residues 1–525), N-terminal region (NTR, residues 1–369) and C-terminal region (CTR, residues 370–525) OLD constructs were cloned into pET21b, introducing a 6xHis tag at the C-terminus. Constructs were transformed into BL21(DE3) cells, grown at 37°C in Terrific Broth to an OD_600_ of 0.7–0.9, and then induced with 0.3 mM IPTG overnight at 19°C. Cells were pelleted, washed with nickel loading buffer (20 mM HEPES pH 7.5, 500 mM NaCl, 30 mM imidazole, 5% glycerol (v/v) and 5 mM β-mercaptoethanol), and pelleted a second time. Pellets were typically frozen in liquid nitrogen and stored at −80°C.

Thawed 500 ml pellets of Ts OLD constructs were resuspended in 30 ml of nickel loading buffer supplemented with 3 mg DNase, 10 mM MgCl_2_, 10 mM PMSF and a Roche complete protease inhibitor cocktail tablet. Lysozyme was added to a concentration of 1 mg/ml and the mixture was incubated for 10 min rocking at 4°C. Cells were disrupted by sonication and the lysate was cleared via centrifugation at 13 000 rpm (19 685 g) for 30 min at 4°C. The lysate was then heated at 65°C for 15 min to precipitate heat-labile proteins. Precipitation was removed by centrifugation at 13 000 rpm (19 685 g) for 15 min at 4°C. The supernatant was filtered, loaded onto a 5 ml HiTrap chelating column charged with NiSO_4_, and then washed with nickel loading buffer. Ts OLD proteins were eluted by an imidazole gradient from 30 mM to 1 M. Pooled fractions were dialyzed overnight at 4°C into Q loading buffer (20 mM Tris pH 8, 50 mM NaCl, 1 mM EDTA, 5% glycerol (v/v) and 1 mM DTT). The dialyzed sample was applied to 5 ml HiTrap Q column equilibrated with Q loading buffer, washed in the same buffer and eluted with a NaCl gradient from 50 mM to 1 M. Peak fractions were pooled, concentrated and further purified by size exclusion chromatography (SEC) using either a Superdex 200 16/600 pg column (FL construct) or a Superdex 75 16/600 pg column (NTR and CTR constructs). Native Ts OLD was exchanged into a final buffer of 20 mM HEPES pH 7.5, 150 mM KCl, 5 mM MgCl_2_ and 1 mM DTT during SEC and concentrated to 20–30 mg/ml. Point mutations were introduced by Quikchange (Agilent) and all mutant and truncated Ts OLD constructs were purified in an identical manner as the wildtype protein.

### Inductively coupled plasma atomic emission spectroscopy (ICP-AES)

Ts^CTR^ was cloned into the expression vector pASK-IBA3C, introducing a C-terminal Strep-II tag. Ts^CTR^ was transformed into BL21(DE3) cells, grown at 37°C in Terrific Broth to an OD_600_ of 0.7–0.9, and then induced with 0.3 mM IPTG overnight at 19°C. Cells were harvested and washed in Strep buffer (100 mM Tris–HCl pH 8, 500 mM NaCl, 5 mM β-mercaptoethanol). Pellets were resuspended in 50 ml of Strep buffer supplemented with 3 mg DNase, 2 mM MgCl_2_, 10 mM PMSF, a Roche complete protease inhibitor cocktail tablet, and 1 mg/ml lysozyme. Following a 10 min incubation at room temperature, the cells were sonicated and cleared via centrifugation. The supernatant was filtered, loaded onto a 5 ml StrepTrap column, and washed with Strep buffer. The protein was eluted with Strep buffer supplemented with 2.5 mM d-desthiobiotin. The protein was pooled, concentrated, and injected onto a Superdex 75 10/300 GL column. Ts^CTR^ was exchanged into a final buffer of 20 mM HEPES pH 7.5 and 50 mM NaCl that was first passed through Chelex 100 resin to remove contaminating divalent cations. The final protein sample was concentrated to ∼10 mg/ml. Approximately 500 μl the protein sample was dried under vacuum and resuspended in 10 ml of 2% nitric acid. Samples were analyzed with an iCAP 6000 ICP-ES (Thermo). Measurements were done in triplicate. The determined milliequivalents of metal per protein molecule are listed in Supplementary Table S1.

### Size exclusion chromatography coupled to multiangle light scattering

The oligomeric state of Ts OLD was determined by SEC coupled to multiangle light scattering (SEC-MALS). Ts^FL^, Ts^NTR^ and Ts^CTR^ constructs were all loaded at 4 mg/ml onto a Superdex 200 10/300 Increase column (GE) in size exclusion buffer (20 mM HEPES pH 7.5, 150 mM KCl, 5 mM MgCl_2_ and 1 mM DTT) at a flow rate of 0.7 ml/min. Eluent from the sizing column flowed directly to a static 18-angle light scattering detector (DAWN HELEOS-II) and a refractive index detector (Optilan T-rEX) (Wyatt Technology) with data collected every second. Molar mass was determined using the ASTRA VI software. Monomeric BSA (Sigma) was used for normalization of light scattering.

### Crystallization, X-ray data collection, and structure determination

Native Ts^FL^ at 10 mg/ml was crystallized by sitting drop vapor diffusion at 20°C in 0.1 M NaOAc pH 5.8, 0.3 M AmSO_4_, 7% PEG MME 2000 and 5 mM SmCl_3_ or PrCl_3_. Crystals were of the space group *I*2_1_2_1_2_1_ with unit cell dimensions *a* = 83.5 Å, *b* = 101.9 Å, *c* = 203.1 Å and α = 90°, β = 90°, γ = 120° and contained a monomer in the asymmetric unit. Heavy metal derivitization was carried out in a solution of 10% PEG MME 2000 and 0.1 M NaOAc pH 5.8. Crystals were soaked in stabilizing solution containing 10 mM potassium tetracyanoplatinate (K_2_Pt(CN)_4_) for 1 h. After soaking, samples were cryoprotected by transferring the crystal directly to Parabar 10312 (Hampton Research) prior to freezing in liquid nitrogen. Crystals were screened and optimized using the NE-CAT 24-ID-C and 24-ID-E beamlines. Single-wavelength anomalous diffraction (SAD) data (8) were collected remotely on the tuneable NE-CAT 24-ID-C beamline at the Advanced Photon Source at the platinum edge (λ = 1.0718 Å) at 100 K to a resolution of 2.12 Å (Supplementary Table S2). Data were integrated and scaled via the NE-CAT RAPD pipeline, employing XDS (9) and AIMLESS (10) for data integration and scaling respectively. A total of three platinum sites were found using SHELX (11) and used for initial phasing. Density modification and initial model building was carried out using the Autobuild routines of the PHENIX package (12). Further model building and refinement was carried out manually in COOT (13) and PHENIX (12). Given the poor quality of the initial data in the highest resolution shell, data was truncated to 2.20 Å during model building and refinement to achieve a CC_1/2_ of 60% in the highest resolution shell. The resulting model was refined with *R*_work_/*R*_free_ values of 20.4%/23.8% (Supplementary Table S2). During refinement we determined that one of the platinum sites identified during phasing was occupied by a samarium atom. The final model contained residues 1–142, 149–237 and 245–525, 66 water molecules, two platinum atoms, one samarium atom, eight sulfate ions and three HEPES molecules.

Structural superpositions were carried out in Chimera (14) and conservation based coloring was generated using the ConSurf server (15). All structural models were rendered using Pymol (Schrodinger).

### DNA cleavage assays

100 ng of lambda genomic DNA or pUC19 plasmid was incubated with 4 μM protein in a final volume of 20 μl in DNA cleavage buffer (20 mM Tris–OAc pH 7.9, 50 mM K-OAc, 0.1 mg/ml BSA and 10 mM divalent metal as chloride salts). Contaminating metals were removed from DNA cleavage buffer with Chelex 100 prior to adding the metal or metals of interest. Reactions were carried out at 65°C for 5 or 15 min and then quenched with 5 μl of 0.5 M EDTA pH 8.0. Samples were analyzed via native agarose electrophoresis. The signal of the initial DNA substrate (intact lambda DNA or supercoiled plasmid) was measured and quantified using the BioRad Image Lab software package. To determine the amount of degraded DNA, the intensity of the intact lambda DNA or supercoiled plasmid in each lane was divided by the signal intensity of intact DNA in the protein-free condition. The quantification bar graphs represent the average of three independent trials with error bars representing the standard error of the mean. Mutant constructs were assayed in the presence of 10 mM MgCl_2_ and 1 mM CaCl_2_.

### Exonuclease assays

The following DNA oligonucleotides for exonuclease assays were synthesized commercially by Integrated DNA Technologies (IDT):

Exo_70_US (5′ or 3′ labeled with 6-carboxyfluorescein, 6-FAM)

5′- ACT GAA GTT CTC CTC GGG TCA TAA TTG TAC TGA CGA CAG CCG TGT TCC CGG TTT CTT CAG AGG CCA CAG AGG GAT-3′

Exo_70_LS

5′- ATC CCT CTG TGG CCT CTG AAG AAA CCG GGA ACA CGG CTG TCG TCA GTA CAA TTA TGA CCC GAG GAG AAC TTC AGT-3′

Lyophilized single-stranded oligonucleotides were resuspended to 1 mM in 10 mM Tris–HCl and 1 mM EDTA and stored at −20°C until needed. Duplex substrates were prepared by heating equimolar concentrations of complementary strands (denoted with suffixes ‘us’ and ‘ls’ indicating upper and lower strands) to 95°C for 15 min followed by cooling to room temperature overnight. Two substrates (5′ or 3′ 6FAM-labeled Exo_70_US each with Exo_70_LS) were prepared. For each substrate, a 150 μl reaction containing 4 μM protein and 75 pmol of labeled double stranded DNA was prepared in exonuclease buffer (20 mM Tris–OAc pH 7.9, 50 mM K-OAc, 0.1 mg/ml BSA, 10 mM MgCl_2_ and 1 mM CaCl_2_) and incubated at 65°C. 20 μl aliquots were taken at the indicated timepoints and quenched with 3× loading buffer (80% formamide and 1× TBE). Samples were analyzed by a denaturing (8 M urea) 14% polyacrylamide gel and visualized using Bio-Rad ChemiDoc XRS+.

### ATPase assays

ATPase activity was characterized using a colorimetric Malachite green assay that monitored the amount of free phosphate released over time (16). The temperature dependence of Ts OLD ATPase activity was carried out in ATPase reaction buffer (20 mM Tris–HCl pH 8, 50 mM NaCl, 5 mM MgCl_2_) with 1 mM ATP and 4 μM Ts OLD. At each time point, 20 μl samples were taken and quenched with 5 μl of 0.5M ETDA pH 8.0. 150 μl of filtered Malachite green solution (16) were added to each sample and incubated for 5 min. Samples were then visualized in a Thermo Multiskan GO spectrophotometer at 650 nm. Rates were plotted in KaleidaGraph (Synergy Software). All ATPase mutant assays were carried out in ATPase reaction buffer with the 0.5 mM ATP and 4μM protein at 65°C. A single-stranded (unlabeled Exo_70_US, see above) or double-stranded (annealed unlabeled Exo_70_US and Exo_70_LS, see above) 70-mer DNA substrate was added at 8 μM in ATPase reaction buffer with 0.5 mM ATP and 4 μM protein at 65°C to assess whether ATPase activity is stimulated by DNA.

### *In vivo* characterization of P2 *old* mutants

Electrocompetent cells of *E. coli* strain SK129 (17) were transformed with the pBAD vector carrying either wildtype or mutant P2 *old* genes and allowed to recover at 30°C to ensure wildtype RecBC function. Transformed cells were grown overnight at 30°C and serial 10-fold dilutions of the cultures were then spotted onto LB agar supplemented with either 0.1% arabinose or 0.1% glucose. Plates were incubated overnight at 37°C to ensure reduction of RecBC function. Colony-forming units (cfu) were counted at the dilution yielding the highest number of discrete colonies. Based on the dilution, this number was converted to reflect the cfu/ml of the undiluted bacterial culture. Relative viability of the strains was determined by comparing the cfu/ml of the induced (arabinose) over the repressed (glucose) conditions. The quantification of these data was the result of four independent trials. When induced, wildtype and D541A mutant of P2 OLD caused a severe growth defect in in the SK129 cells at 37°C, requiring ∼10× magnification to accurately assess the number of surviving colonies.

## RESULTS

### Ts OLD functions as a metal-dependent nuclease *in vitro*

**Figure 1.**
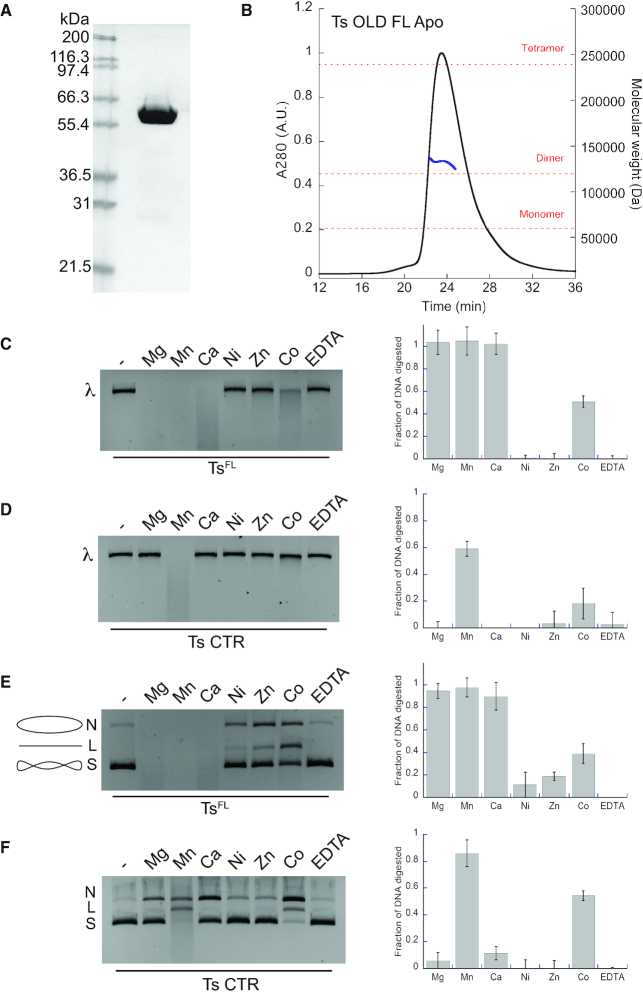
Purification and biochemical characterization of Ts OLD. (**A**) SDS-PAGE gel showing purified full-length Ts OLD (Ts^FL^). (**B**) SEC-MALS analysis of Ts^FL^. UV trace (black) and calculated molecular weight based on light scattering (blue) are shown. (**C**) Metal-dependent nuclease activity of Ts^FL^ on linear λ DNA with quantification of DNA digestion. (**D**) Metal-dependent nuclease activity of Ts OLD C-terminal region (Ts^CTR^) on linear λ DNA. (**E**) Metal-dependent nuclease activity of Ts^FL^ on supercoiled pUC19 DNA. N’, ‘L’ and ‘S’ denote the positions of ‘nicked’, ‘linearized’, and ‘supercoiled’ DNA respectively. DNA topology is also illustrated by the small cartoons to the left of the gel. (**F**) Metal-dependent nuclease activity of Ts OLD C-terminal region (Ts^CTR^) on supercoiled pUC19 DNA. Graphs represent the average of three independent trials with error bars representing the standard error of the mean.

To elucidate the architecture and function of the Class I OLD proteins, we screened a multitude of homologs to find a suitable candidate for structural and biochemical studies. Of these, only the thermophilic *T. scotoductus* showed high levels of expression in *E. coli* and produced protein that remained soluble throughout purification (Figure 1A). Size exclusion chromatography coupled to multi-angle light scattering (SEC-MALS) revealed that full-length Ts OLD (Ts^FL^) forms stable dimers in solution, regardless of the nucleotide state (Figure 1B, Supplementary Figure S1A and B). The N-terminal region encompassing the ATPase domain (NTR, residues 1–369) also dimerizes on its own (Supplementary Figure S1C) while the CTR containing the Toprim domain (residues 370–525) remains monomeric (Supplementary Figure S1D), consistent with the oligomeric state of Class 2 OLD CTRs (3). These data suggest that the structural features mediating OLD nuclease dimerization reside in the NTR and do not require ATP binding or hydrolysis.

Toprim family enzymes catalyze metal-dependent nicking and cleavage of nucleic acid substrates (18). To determine if Ts OLD acts similarly *in vitro*, we incubated Ts^FL^ with linearized λ phage DNA in the presence of different divalent cations and monitored the disappearance of the substrate as nuclease activity was activated over the course of 15 min (Figure 1C). Ts^FL^ exhibits rapid DNA degradation activity on linear DNA in the presence of Mg^2+^, Mn^2+^ and Ca^2+^, digesting ∼100% of the input DNA. Weaker activity is observed in the presence of Co^2+^ (51% digested) with little to no activity in the presence of Ni^2+^ or Zn^2+^. When incubated for only five minutes, Ts^FL^ is most strongly stimulated by Mn^2+^, digesting 26% of the substrate compared to <10% digested with Mg^2+^, Ca^2+^ and Co^2+^ (Supplementary S1E). The Toprim-containing Ts^CTR^, in contrast, shows a strong preference for Mn^2+^, digesting 74% of the lambda DNA, and weaker activity with Co^2+^ (19%) (Figure 1D). Other metals did not stimulate Ts^CTR^ DNase activity. The degradation of DNA in the presence of Ts^CTR^ alone suggests that the catalytic machinery responsible for nuclease cleavage is localized to the C-terminal region of Ts OLD as was also observed in the OLD homologs from *Xanthomonas campestris* pv. *campestris* and *Burkholderia pseudomallei* (3).

To characterize nuclease activity further, we assessed how Ts OLD acts on circular plasmids. We incubated Ts^FL^ with supercoiled pUC19 DNA (S) for 15 min in the presence of different divalent metals and measured activity by the disappearance of DNA signal and by the appearance of slower migrating bands as the substrate was nicked (N) and linearized (L) by the enzyme (Figure 1E). Ts^FL^ can nick and partially linearize supercoiled substrates with Co^2+^, Zn^2+^ and Ni^2+^ and efficiently degrades circular substrates with Mg^2+^ (94% digested DNA), Mn^2+^ (97%) and Ca^2+^ (89%). This degradation occurs rapidly over the course of this experiment, evidenced by the absence of any observable linearized intermediate. Again, a shorter incubation period shows Mn^2+^ has the strongest stimulating effect, degrading ∼80% of the supercoiled plasmid in five minutes (Supplementary Figure S1F). Ts^CTR^ nicks plasmid DNA with Mg^2+^, Mn^2+^, Ca^2+^, and Co^2+^, with a linearized band visible with Mn^2+^ and Co^2+^ (Figure 1F). Significant degradation of circular DNA by Ts^CTR^ is only observed in the presence of Mn^2+^ where 89% of the plasmid DNA is degraded (Figure 1F).

Although Class 2 OLD nucleases can use a variety of metals to catalyze nuclease cleavage *in vitro*, inductively coupled plasma atomic emission spectroscopy (ICP-AES) revealed that purified Bp and Xcc OLD proteins preferentially contain bound Ca^2+^ and Mg^2+^ (3). Moreover, calcium potentiates the metal-dependent nuclease activity of these proteins *in vitro* when added in the presence of magnesium (3). To test whether Ts OLD exhibits the same metal-binding properties, we analyzed purified Ts^CTR^ by ICP-AES. As with Class 2 enzymes, calcium and magnesium were the most abundant metals present with some nickel and trace amounts of zinc (Supplementary Table S1). No manganese or cobalt was detected. Given these observations, we assessed Ts OLD nuclease function with different mixtures of calcium and magnesium. At short incubations of 5 min at 65°C, we observe a robust stimulation of Ts^FL^’s nuclease activity on both linear and supercoiled substrates when increasing amounts of CaCl_2_ are added to the enzyme with 10 mM MgCl_2_ (Supplementary Figure S2A and B). This stimulation reaches a maximum at a concentration of 5 mM CaCl_2_. The catalytic enhancement due to supplemental calcium is even more pronounced at longer time points, as every concentration added promotes the near complete degradation of DNA (Supplementary Figure S2C and D). Importantly, the combination of magnesium and calcium is more efficient at catalyzing substrate degradation than either metal alone under all conditions tested (Supplementary Figure S2). These results suggest that calcium is important for the function of both Class 1 and Class 2 OLD proteins.

The nuclease activity of the Class 1 OLD protein from bacteriophage P2 was previously shown to be stimulated *in vitro* by the presence of ATP (6). We therefore tested whether Ts OLD responded similarly. The cleavage profile of Ts^FL^ on both linear and supercoiled DNA substrates remained largely unchanged with the addition of either ATP or the nonhydrolyzable analog AMP-PNP (Supplementary Figure S3A–D). ATP at high concentration (1mM) partially inhibits the degradation of supercoiled plasmid DNA (Supplementary Figure S3B). This modest effect, however, may be indirect due to metal chelation by ATP. These data argue that the nuclease activity of Ts OLD is not directly dependent on ATP binding and/or hydrolysis and is primarily driven by the CTR.

Although our assays demonstrate Ts OLD can degrade linear substrates efficiently under a variety of conditions, they do not specifically differentiate between exo- or endonuclease activities. To evaluate the exonuclease function of Ts^FL^, we assessed the degradation of 5′- and 3′-6-carboxyfluorescein (6-FAM)-labeled DNA substrates. Ts^FL^ degrades the 3′-labeled substrate while there is no apparent degradation of the 5′ labeled substrate (Supplementary Figure S3E). This suggests that Ts^FL^ can act as an exonuclease that degrades DNA from 5′-3′ and that the 5′ 6-FAM label likely blocks Ts^FL^ from accessing that end of the molecule. Importantly, this directionality is consistent with nuclease functions of P2 OLD (6) and the Class 2 OLD homolog from *Burkholderia pseudomallei* (3).

### Structural organization of Ts OLD

**Figure 2.**
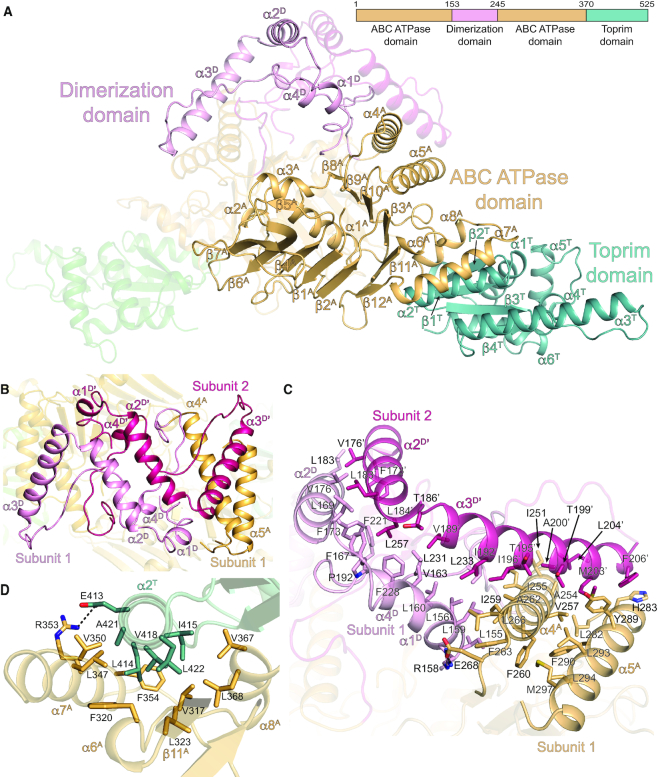
Architectural organization of full-length Ts OLD. (**A**) Structure of Ts OLD dimer. Coloring is as follows: ATPase domain orange; dimerization domain, light pink; Toprim domain, teal. Dimer is oriented orthogonal to the two-fold axis with one subunit rendered transparent for clarity. Cartoon of domain the architecture with numbered boundaries is included on the right. Secondary structure elements are numbered sequentially within each domain with the superscripts ‘A’, ‘D’, and ‘T’ denoting the ATPase, dimerization, and Toprim domains respectively (See Supplementary Figure S5A). (**B**) Top down view of dimerization domain helices. Helices from Subunit 1 and Subunit 2 are colored light pink and warm pink respectively. (**C**) Dimerization domain interactions. Residues contributing to the dimerization interface are shown as sticks and labeled. (**D**) Interactions stabilizing the ATPase-Toprim interface. Contributing side chains are shown as sticks and labeled. Dashed black line denotes salt bridge between R353 and E413.

Optimized crystals of native Ts^FL^ routinely diffracted beyond 2.5 Å and structure was solved by SAD phasing using a platinum soaked derivative. The resulting model was refined to 2.20 Å and contained residues 1–142, 149–237 and 245–525 (Supplementary Table S2). Consistent with our SEC-MALS data, symmetry-related molecules form dimers in crystal lattice (Supplementary Figure S4A). Ts^FL^ subunits are comprised of three structural domains: an N-terminal ATPase domain, a helical dimerization domain, and a C-terminal Toprim domain (Figure 2). The ATPase domain core consists of 12 β-strands organized into an eleven stranded β-sheet ordered β7-6-4-1-2-12-11-3-10-9-8 with the β5 strand packing against the end of the extended β6 strand (Figure 2A, Supplementary Figure S5A). The sheet folds at the nexus of β2 and β12, creating two segments that sandwich a central alpha helix (α1^A^). Helices α2^A^ and α3^A^ flank the sandwich, capping the end of α1^A^. The remaining five helices line the exterior, with two (α4^A^ and α5^A^) contacting the dimerization domain of the opposite subunit *in trans* (Figure 2A–C) and three (α6^A^, α7^A^ and α8^A^) interacting with the Toprim domain *in cis* (Figure 2A and D). The dimerization domain inserts between the β8 and β9 strands of the ATPase domain and consists of four helices (α1^D^, α2^D^, α3^D^ and α4^D^) that form a kinked structure (Figure 2B, Supplementary Figure S5A). α1^D^, α2^D^ and α4^D^ from each subunit directly associate *in trans* at the dimer interface while α3^D^ bends back and rests on top of α4^A^ and α5^A^ (Figure 2B). Together these interactions form an extensive hydrophobic network (Figure 2C) that buries 3479 Å^2^ of surface area and maintains the tight association between the subunits.

Each Ts OLD Toprim domain contains a canonical four-stranded parallel β-sheet (β2^T^–β1^T^–β3^T^–β4^T^) surrounded by three α-helices (α1^T^, α2^T^ and α3^T^) (Figure 2A, Supplementary Figure S5). As observed in Class 2 OLD nucleases (3), the orientations of α2 and α3 are shifted relative to other Toprim domains (Supplementary Figure S5B). Ts OLD, however, lacks an Insert 3 helix and contains three additional α-helices following the terminal β4^T^ strand (Supplementary Figure S5A and S5C). The amphipathic α2^T^ helix packs against a hydrophobic groove on the edge of ATPase domain formed by α6^A^, α7^A^, α8^A^ and β11^A^ to bury 920Å^2^ (Figure 2D). The Toprim residues at this interface are highly conserved among Class 1 homologs (Supplementary Figure S6 and S7A), suggesting this interaction is maintained throughout the family. A single salt bridge between R353 and E413 adds additional stabilization. The Toprim domains do not participate in dimerization, instead extending outward nearly orthogonal to the 2-fold symmetry axis (Figure 2A).

### Ts^FL^ identifies the determinants of OLD ATP hydrolysis

**Figure 3.**
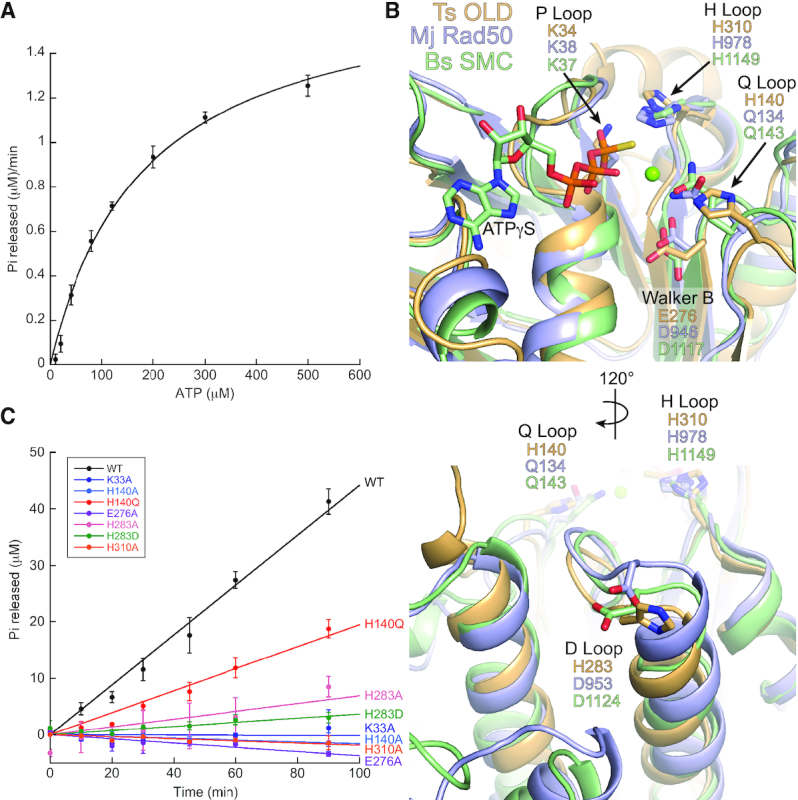
Catalytic determinants of Ts OLD ATP hydrolysis. (**A**) Michaelis–Menten kinetics of Ts^FL^. (**B**) Structural superposition of the ATPase active site loops from Ts OLD (light orange), *Methanococcus jannashii* Rad50 (light blue; PDB: 5DNY), and *Bacillus subtilis* SMC (light green; PDB: 5H66). P loop, Walker B, Q loop, D loop and H loop are labeled with the identity and position of the catalytic residues for each protein species. (**C**) Initial ATP hydrolysis rates of wildtype (WT) Ts OLD compared to mutants with single alanine substitutions for each of the residues involved in ATP binding and hydrolysis. Data points are the average of three independent trials with error bars representing the standard error of the mean.

Ts OLD ATP hydrolysis is strongly dependent on temperature, with an optimal activity at 65°C that reflects its thermophilic nature (Supplementary Figure S8A). We observe a modest basal turnover at this temperature with a *k*_cat_ of 0.44 ± 0.3 min^−1^ and *K*_m_ of 180 ± 26 μM (Figure 3A). Addition of either ss- or dsDNA stimulates the basal hydrolysis rate by ∼3.5-fold (Supplementary Figure S8B). Despite attempts to co-crystallize with ATP analogs, only a sulfate ion deriving from the crystallization buffer was present in the Ts^FL^ active site (Supplementary Figure S4B). The sulfate binds in the position normally occupied by the β-phosphate of ATP (Supplementary Figure S4C) and forms stabilizing hydrogen bonds with the main chain atoms of G31, G33 and K34 and the side chain hydroxyl of T35 (Supplementary Figure S4B). Q59 from the adjacent subunit reaches into the pocket to provide an additional hydrogen bond (Supplementary Figure S4B). Two platinum ions also occupy a site adjacent to the P loop (Supplementary Figure S4B), a consequence of heavy atom soaking. One localizes where the 4′-carbon of the ribose ring typically sits and is coordinated by the sulfate and the T36 side chain (Supplementary Figures S4B and C). The second forms water mediated interactions with R11 and T36 (Supplementary Figure S4B). The DALI alignment algorithm (19) identifies *Methanococcus jannaschii* (Mj) Rad50 (PDB: 5DNY, *Z*-score = 17.5, RMSD = 3.7) and the *Pyrococcus furiosus* (Pf) Smc-ScpAB complex (PDB: 4I99, *Z*-score = 8.2, RMSD = 3.8) as the nearest structural homologs, suggesting Ts OLD may be related to the SMC/Rad50/RecN/RecF family of DNA repair **A**TP-**b**inding **c**assette (ABC) proteins (20). Structural superposition confirms their overall similarity (Supplementary Figure S9) and identifies residues in Ts OLD that contribute to ATP binding and hydrolysis (Figure 3B).

ABC ATPases share a RecA-like nucleotide binding domain (NBD) that dimerizes upon activation, sandwiching two ATP molecules at the interface between the associating subunits (21). Six conserved sequence motifs—P loop/Walker A, Walker B, the ABC signature sequence, Q loop, D loop and H loop—contribute to nucleotide binding and hydrolysis (Supplementary Figure S10). As in other ATPases, the P loop/Walker A motif coordinates the α- and β-phosphates of ATP while the Walker B motif stabilizes an essential magnesium cofactor and coordinates the nucleophilic water (22). The Q loop also acts *in cis* to coordinate the magnesium and catalytic water, helping to sense the nucleotide state and orchestrate associated conformational changes (23,24). A conserved histidine in the H loop balances the negative charge of the aspartate/glutamate in Walker B and the γ-phosphate in the transition state (25–27). The signature sequence (LSGGQ) characteristic of all ABC proteins and the D loop both act *in trans*, where the former contacts the nucleotide and γ-phosphate of the adjacent subunit and the latter serves a dual function in positioning the nucleophilic water molecule and stabilizing the Walker A motif (28–30).

Ts OLD contains a number of sequence variations across these catalytic motifs compared to the SMC/Rad50/RecN/RecF family ABC proteins (Figure 3B, Supplementary Figure S10B). Although K34 and H310 structurally align with conserved P loop and H loop side chains, a glutamate (E276) replaces the Walker B aspartate and histidines H140 and H283 substitute for the conserved glutamine and aspartate side chains in the Q loop and D loop, respectively. These deviations from canonical ABC consensus sites vary in their conservation across other Class 1 OLD homologs (Supplementary Figure S6). For example, H140 in the Q loop is poorly conserved, with many species having an arginine at this position. Exchange of an asparagine for H310 in the H loop also occurs at a similar frequency. The remaining residues are largely conserved. Notably, the ABC signature sequence is degenerate in all Class 1 OLD homologs and also absent from the Class 2 homolog from *Burkholderia pseudomallei* (Supplementary Figures S6 and S10B).

To elucidate the functional relevance of this putative catalytic machinery, we mutated each residue individually to alanine and tested the effect on ATP hydrolysis. The K34A, H140A, E276A and H310A substitutions each completely abolish ATPase activity (Figure 3C), underscoring their crucial catalytic function. Mutating the Q loop histidine to the corresponding canonical glutamine (H140Q) partially restores catalytic turnover (2.4-fold decrease relative to wildtype). The D loop mutation H283A decreases the hydrolysis rate ∼4-fold, suggesting a moderate contribution to the enzymatic activity. Substitution of an aspartate in this position as in canonical ABC ATPases does not improve the hydrolysis rate, underscoring the unique structural and enzymatic properties of Ts OLD. ATPase mutants have no measurable effect on nuclease function *in vitro* (Supplementary Figure S8C and D), consistent with our findings that DNA cleavage activity is not directly dependent on ATP binding and/or hydrolysis (Supplementary Figure S3A–D). Together these data provide new insights into the machinery governing OLD family ATPase activity.

### Ts OLD ATPase domains adopt a non-productive conformation

**Figure 4.**
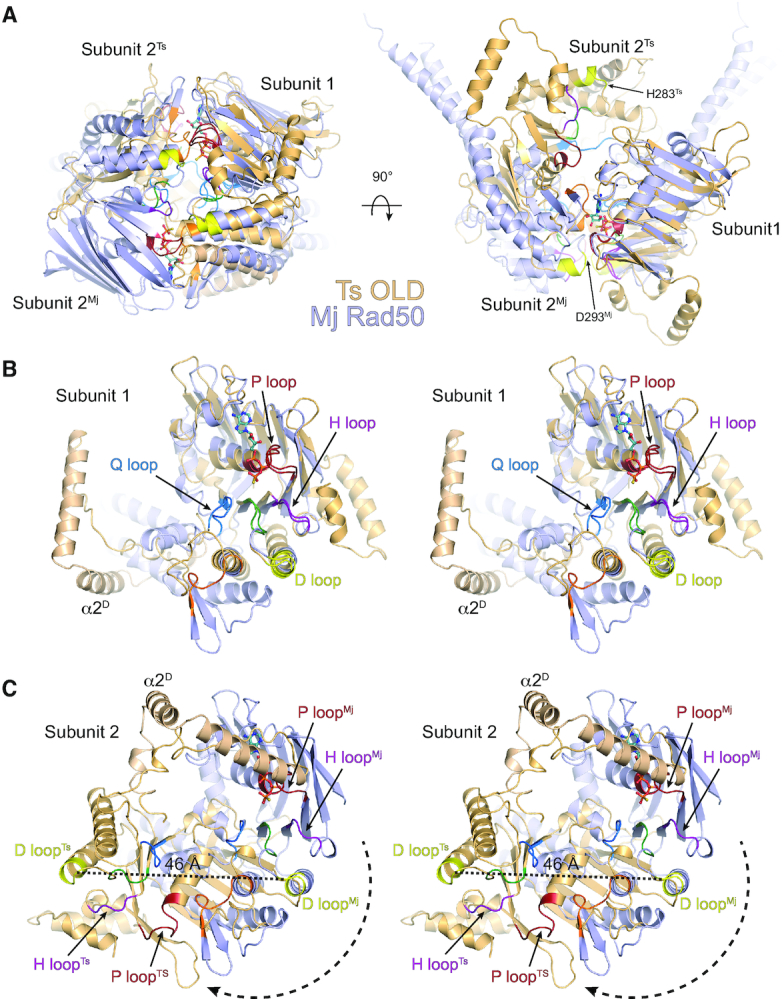
Ts OLD ATPase domains adopt a non-productive conformation. (**A**) Structural superposition of the ATPase domain dimers from Ts OLD (light orange) and *Methanococcus jannashii* (Mj) Rad50 (light blue; PDB: 5DNY). Bottom (left) and side (left) views are depicted. Aligned domains are labeled as ‘Subunit 1’ with the second domain in each structure labeled as ‘Subunit 2’ to highlight alternative conformations. Mj Rad50 adopts the canonical, head-to-head dimer arrangement necessary for productive hydrolysis in ABC ATPases (See Supplementary Figures S10 and S11). Catalytic motifs in each structure are colored as follows: P loop, red; Q loop, blue; Walker B, green; D loop, yellow; ABC signature sequence, orange; H loop, purple. Arrows mark the Cα positions of the *trans*-acting catalytic D loop residues (H283 in Ts OLD; D293 in Mj Rad50) in Subunit 2 of each structure. (**B**) Stereo view of the aligned ATPase domains (Subunit 1) from dimer superposition in A. The P loop, Q loop, D loop, and H loop are labeled. Position of the α2^D^ helix in Ts OLD is marked for reference. (**C**) Stereo view of non-aligned ATPase domains (Subunit 2) from dimer superposition in A. Subunits are shown in the same orientation as in B. Relative positions of the P loop, D loop, and H loop are labeled in each structure. Position of the α2^D^ helix in Ts OLD is marked for reference. Dashed black arrow indicates rotation of the second Ts ATPase domain relative second subunit Mj Rad50, which adopts a hydrolysis-competent conformation. Dashed black line indicates displacement (measured from Cα to Cα) of the *trans-*acting H283 in the Ts D-loop relative to its counterpart (D293) in Mj Rad50.

The crystal structures of different SMC/Rad50/RecN/RecF family proteins show that head-to-head dimerization of the NBDs properly positions the catalytic machinery for productive ATP hydrolysis (Figure 4A, Supplementary Figures 10C and 11A–C) (31–33). In this arrangement, the ABC signature sequence, D loop, and H loop are oriented toward the active site of the opposing subunit in close approach to the P loop and bound nucleotide (Supplementary Figure S10A and C). The organization of the ATPase domains in the nucleotide-free Ts^FL^ dimer differs from this configuration (Supplementary Figure S11D), with the individual ATPase domains more splayed apart and twisted. Superposition of Ts^FL^ with the Mj Rad50 shows that one subunit from each ATPase domain dimer aligns with minimal structural deviations (Figure 4A and B). The second Ts subunit, however, is significantly rotated relative to the dimer axis (Figure 4A and C, dashed arrow). The *trans*-acting catalytic side chain H283 of the Ts OLD D loop is far removed from the composite active site in this arrangement—displaced nearly 46 Å (Cα to Cα) from the respective position of its counterpart (D953) in Mj Rad50 (Figure 4A and C)—and would be unable to exert its functional effects efficiently. This suggests that the Ts^FL^ conformation captured in the crystal is non-productive for ATPase hydrolysis and would require additional conformational rearrangements, likely triggered by nucleotide binding, to achieve a more canonical organization of the ATPase domains.

### OLD proteins share a conserved mechanism for nuclease cleavage

**Figure 5.**
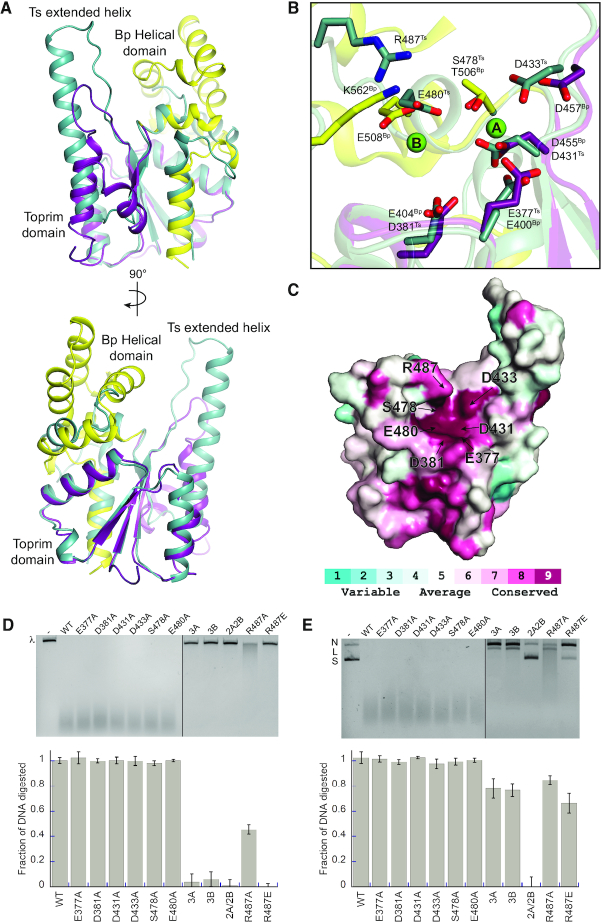
Class 1 and Class 2 OLD proteins share a conserved mechanism for nuclease cleavage. (**A**) Structural superposition of OLD CTR structures from Ts (teal) and *Burkholderia pseudomallei* (Bp, PDB: 6NK8; yellow and purple). (**B**) Zoomed view of active sites identifying catalytic machinery required for nuclease function. Coloring as in A. Bound magnesium ions in Bp^CTR^ are shown as green spheres with ‘A’ and ‘B’ denoting the positions of ‘metal A’ and ‘metal B’ respectively. (**C**) Conservation of active site residues among Class 1 OLD homologs. Coloring generated using the ConSurf server (15) and the alignment in Supplementary Figure S6. (**D**) Cleavage activities of Ts OLD active site mutants on linear λ DNA. Mutant abbreviations are as follows: 3A, E377A/D431A/D433A; 3B, D381A/S478A/E480A; 2A/2B, D431A/D433A/S478A/E480A. (**E**) Nicking and cleavage activities of Ts OLD mutants on supercoiled pUC19 DNA. All cleavage assays were performed in the presence of magnesium and calcium as described in the Materials and Methods. Graphs represent the average of three independent trials with error bars representing the standard error of the mean.

Our previous structural study identified the catalytic machinery of Class 2 OLD proteins and supports a two-metal mechanism for nuclease cleavage (3). Class 1 proteins are smaller and show significant sequence divergence in the C-terminus relative to their Class 2 counterparts, suggesting possible architectural and mechanistic differences. Superposition of the Ts^FL^ and Bp^CTR^ models reveals a high degree of structural similarity between the Toprim core in both proteins (RMSD: 0.558 Å) (Figure 5A). Importantly, the positions of the α2 and α3 helices relative to the central β-sheet remain unchanged between both classes but distinct from every other Toprim containing protein (Supplementary Figure S5B). Ts OLD, however, shows two key structural differences: it lacks both an Insert 3 helix between α3 and β4 that is present in Class 2 OLD proteins and numerous topoisomerases (3) and a C-terminal helical domain (Figure 5A, Supplementary Figures S5 and S6). Despite these topological differences, the critical catalytic side chains responsible for Class 2 nuclease activity are spatially conserved in Ts OLD (Figure 5B). Ts residues E377, D431 and D433 align with the Bp OLD metal A binding residues E400, D455, and D457 while D381, S478, E480 roughly superimpose with the Bp metal B ligands E404, T506 and E508. These residues are conserved among all Class 1 homologs (Figure 5C, Supplementary Figure S6) and in the Ts^FL^ structure coordinate a single samarium ion from the crystallization conditions (Supplementary Figure S4D and E). This samarium binds offset from either of the catalytic metal binding sites (Supplementary Figure S4D), owing to additional crystal packing interactions that complete the coordination sphere *in trans* and perturb the canonical active site organization (Supplementary Figure S4E). Mutation of the putative Ts metal A binding side chains to alanine (3A mutant, E377A/D431A/D433A) decreases degradation of linear and supercoiled DNA by 15- and 12-fold respectively (Figure 5D and E). Similarly mutating the metal B binding residues (3B, D381A/S478A/E480A) leads to a 10- and 20-fold decrease in activity on linear and supercoiled DNA (Figure 5D and E). As with Class 2 homologs (3), metal A and metal B mutants still retain the ability to nick and linearize circular plasmids (Figure 5E); however, mutating both the metal A and metal B binding sites (2A/2B mutant, D431A/D433A/S478A/E480A) completely abolishes any nicking activity (Figure 5E).

K562 also plays a crucial role in Bp OLD nuclease function, where it acts to stabilize the developing negative charge in the transition state of the phosphoryl transfer reaction and/or protonate the leaving group (3). Superposition shows that the guanidinium group of Ts R487 aligns with the amino group of Bp K562. The ariginine sidechain approaches the active site from a different angle, as it resides in the loop preceding α5^T^ instead of a separate helical domain. A conserved arginine or lysine residue is present at this position in all Class 1 OLD homologs (Supplementary Figure S6). To validate the functional importance of this residue, we mutated R487 to either an alanine or glutamate and tested how each affected nuclease activity on either linear or supercoiled DNA. R487E completely abrogates DNA degradation of both substrates while R487A only partially inhibits, limiting degradation of both linear and supercoiled DNA by 3-fold (Figure 5D and E). Together, these data argue that Class 1 and Class 2 OLD nucleases use a conserved set of catalytic machinery and a similar two-metal mechanism to cleave DNA.

### ATPase and nuclease mutations impair P2 OLD activity *in vivo*

**Figure 6.**
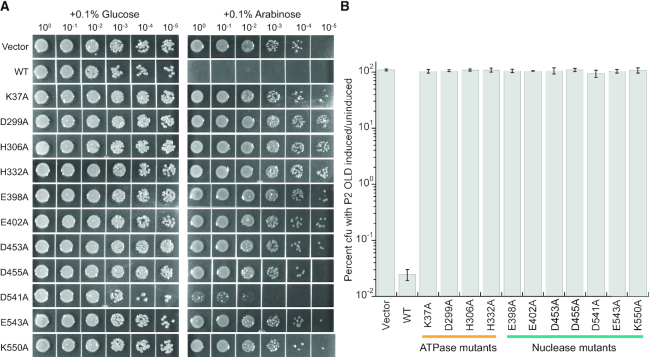
ATPase and nuclease activities are required for P2 OLD function *in vivo*. (**A**) Representative images of spot assay for *E. coli* carrying a temperature-sensitive *recBC*^ts^ allele transformed with arabinose-inducible P2 OLD wild type as well as P2 OLD carrying mutations in the ATPase and nuclease domains. Strains were grown at 37°C overnight, which is not permissive for the RecBC^ts^ function. P2 OLD was induced with 0.1% arabinose or repressed with 0.1% glucose. (**B**) Quantification of colony forming units under P2 OLD induction compared to P2 repression. Graphs represent the average of three independent trials with error bars representing the standard error of the mean.

P2 OLD is a Class 1 enzyme like Ts (3) and has well established functions in bacteriophage defense and survival (4,5). To understand the importance of OLD ATPase and nuclease activities *in vivo*, we leveraged the observation that *old^+^* P2 lysogens kill *E. coli* harboring *recB* and *recC* mutations (4). The *E. coli* strain SK129 contains temperature-sensitive alleles of both *recB* and *recC* such that RecBC function is impaired at 37°C (17). Overexpression of wildtype P2 OLD from an arabinose inducible pBAD vector mimics the phage phenotype and kills this host strain at the 37°C with a cell viability of 0.003% (Figure 6). SK129 colonies surviving under these conditions suffer a severe growth defect. Host killing is supressed at 30°C where RecBC is fully functional (Supplementary Figure S12). SK129 *E. coli* survive and grow normally at either temperature when glucose is substituted for arabinose (Figure 6A and Supplementary Figure S12).

Using this experimental background, we next examined the effect of ATPase and nuclease mutants on P2-mediated host killing. Guided by our structural findings and sequence conservation (Supplementary Figure S6), we identified the putative catalytic side chains in P2 OLD and engineered substitutions in the critical ABC ATPase motifs (K37A, Walker A; D299A, Walker B; H306A, D-loop; H332A, H-loop) and the Toprim metal binding and nuclease residues (E398A, D453A and D455A, metal A; E402, D541 and E543, metal B; K550A). Mutants constructs were cloned into pBAD and overexpressed with arabinose in SK129 *E. coli* at 37°C. Every mutation abolished the lethal effects of P2 OLD expression in the SK129 background and restored the viability at the non-permissive temperature to wildtype levels (Figure 6B). Although D541A rescued viability as measured by cfu/mL (Figure 6B), expression of this mutant causes a noticeable slow growth phenotype (Figure 6A). Together these data indicate that both the ATPase and nuclease activities of P2 OLD are required for function *in vivo* and necessary for host killing when the RecBC recombinational repair pathway is impaired.

## DISCUSSION

Here we described the full-length structure of the Class 1 OLD homolog from *Thermus scotoductus*, which reveals a three domain architecture consisting of an N-terminal ABC ATPase domain, a dimerization domain, and a C-terminal Toprim domain. Our biochemical experiments show that Ts OLD functions as a metal-dependent nuclease *in vitro* and exhibits temperature-dependent ATP hydrolysis, with maximal turnover at 65°C.

The Ts OLD ATPase domain is topologically related to the NBDs of genome maintenance proteins (Supplementary Figure S9). This structural homology allowed us to pinpoint key catalytic residues that are required for ATP hydrolysis (Figure 3B and C) and revealed variations in the canonical ABC sequence motifs that are present in the Ts OLD active site (Supplementary Figure S10B). In particular, we note the absence of a defining ABC signature sequence and the presence of histidines at the positions of the conserved glutamine and aspartate side chains in the Q loop and D loop, respectively. Mutating these histidines to alanines (H140A and H283A) abrogates Ts OLD ATP hydrolysis *in vitro* (Figure 3C). Moreover, substitution of the canonical residues at these positions (H140Q and H283D) fails to restore hydrolysis fully to wildtype levels (Figure 3C). Homologs of the mismatch repair protein MutS contain various substitutions for the glutamine of the Q loop, including a valine in *E. coli* (PDB: 1E3M) and in humans (PDB: 2O8B) and an isoleucine in *T. aquaticus* (PDB: 1EWQ) and *N. gonorrhoeae* (PDB: 5YK4) (34–37). Similarly, the Q loop glutamine is also not conserved in the recombination mediator protein RecF, where arginine (*D. radiodurans*, PDB: 2O5V), valine (*T. thermophiles*, PDB: 5ZWU), and phenylalanine (*C. subterraneus* subsp. *tengcongensis*, PDB: 5Z68) substitutions have been observed (33,38). *D. radiodurans* RecN, in contrast, shows a degenerate D loop sequence (PDB: 4ABY) (Supplementary Figure S10B). Some ABC transporters also show non-canonical active sites including those that can function as asymmetric heterodimers where substitutions render one subunit inactive (39–41). While Ts OLD is not the only ABC-like ATPase to have noncanonical residues in the defining sequence motifs, the variation in its catalytic machinery is a more extreme case. Collectively, these observations argue that the OLD proteins represent a distinct branch of the ABC ATPase superfamily that is most closely related to SMC/Rad50/RecN/RecF DNA repair enzymes.

It is important to note, however, that some of these catalytic residue variants are not strictly conserved among Class 1 OLD nucleases (Supplementary Figure S6). It remains to be seen whether homologs like *Stroptococcus suis*, which has an alanine in place of the histidine in the D loop, retain the ability to hydrolyze ATP. Interestingly, the Class 2 OLD homolog from *Burkholderia pseudomallei* contains a glutamate at the position corresponding to H140 in the Ts Q loop and shows no measurable ATP hydrolysis *in vitro* (Schiltz and Chappie, unpublished data). We do not yet know whether this substitution is directly responsible for the lack of catalytic turnover or whether interactions with other binding partners—including the putative helicase genes that appear in tandem with Class 2 OLD homologs—stimulate the hydrolysis in some way. Further biochemical characterization of representatives from both Classes will be necessary to tweeze out subtle mechanistic differences.

Dimerization of the nucleotide binding domains and subsequent ATP hydrolysis triggers concomitant conformational changes that regulate the biological function of ABC ATPases. The cycle of ATP binding, hydrolysis, and release of ADP controls conformational rearrangements in ABC transporters that pump substrates into or out of cells (41). For many recombination and repair proteins, ATPase activity is coupled to nucleic acid substrate recognition and the licensing of nicking and/or cleavage activities that initiate downstream events (33,42–44). Ts^FL^ dimerizes both in solution (Figure 1B, Supplementary Figure S1) and in the crystal lattice (Supplementary Figure S4A), with the extensive hydrophobic interface between dimerization domain helices providing the primary stabilizing interactions (Figure 2C). While extended helical coils help to tether subunits in many repair family ABC proteins, productive ATP hydrolysis requires a head-to-head engagement of the NBDs to orient critical catalytic motifs *in cis* and *in trans* (Supplementary Figure S10 and S11). The orientation of the ATPase domains in Ts^FL^ is not compatible with this arrangement, as one subunit is significantly rotated (Figure 4). The *trans*-acting H283 of the Ts OLD D loop is displaced from composite active side in this orientation, suggesting our structure has trapped a conformation that is non-productive for ATP hydrolysis. Whether this is a consequence of crystallization or represents a nucleotide-free intermediate that is propagated during the hydrolysis reaction cycle remains to be seen. This does, however, imply that Ts OLD must undergo a major conformational reorganization to achieve productive turnover. Further structural characterization of Ts OLD with different nucleotide analogs will clarify these discrepancies.

In each full-length Ts OLD subunit, the amphipathic α2^T^ Toprim helix packs against the hydrophobic groove in the ATPase domain created by the α6^A^, α7^A^, α8^A^ and β11^A^ (Figure 2D). The engaged surface along the backside of the Toprim is highly conserved not only among Class 1 enzymes but also among Class 2 homologs (Supplementary Figure S7). Although we currently lack a full-length model of a Class 2 OLD protein, we predict that the Toprim and ATPase domains will associate in the same manner among these homologs. Elucidating structural differences between the two classes will aid in our understanding of why Class 2 genes appear in tandem with a specific UvrD/PcrA/Rep-like helicase and whether direct interaction with this helicase is necessary for function.

The CTRs of Class 2 OLD homologs consist of a Toprim domain with altered architecture and a unique helical domain. Conserved side chains in both domains contribute to the nuclease active site and adopt a geometry that supports a two-metal catalysis mechanism for cleavage (3). Ts OLD shares the altered Toprim domain (Supplementary Figure S5B) but lacks the C-terminal helical bundle (Figure 5A, Supplementary Figure S5). This key difference explains our inability to identify the catalytic residues of Class 1 enzymes by sequence alignment alone. Structural superposition, however, shows that Ts OLD retains the critical metal A and metal B binding residues and has an arginine that functions like K562 in Bp OLD, presumably stabilizing the developing negative charge in the transition state of the phosphoryl transfer reaction and/or protonating the leaving group (Figure 5B). These residues are conserved among Class 1 homologs (Figure 5C, Supplementary Figure S6), suggesting all OLD family nucleases use a conserved set of catalytic machinery. Our biochemical data support this idea, as metal A and metal B mutants produce similar DNA nicking and cleavage defects in both Ts OLD (Figure 5D and E) and Class 2 enzymes (3).

ICP-AES analysis indicates that calcium and magnesium are the preferred metals associated with purified Ts OLD (Supplementary Table S1). Consistent with this, Ts OLD nuclease activity is stimulated in the presence of both magnesium and calcium (Supplementary Figure S2). The purified Class 2 OLD homologs from *Burkholderia pseudomallei* and *Xanthomonas campestris* pv. *campestris* show a similar binding preference for calcium and magnesium by ICP-AES and also display stimulated nuclease activity when the two metals are mixed together (3). These properties thus appear to be another defining characteristic of the OLD proteins.

Our structural studies represent a major advance in understanding the enzymatic functions of OLD proteins. Despite the novel insights we provide regarding the underlying catalytic mechanisms and machinery, we still know very little about OLD function *in vivo*. Much of our current knowledge derives phage genetics, which established that the P2 *old* gene is dominant, interferes with normal bacteriophage λ growth when present in lysogens, and mediates killing of so-called ‘lysogenization defective’ *E. coli* hosts that are impaired in recombinational repair (4,5). Here were have demonstrated that P2-mediated killing of these *E. coli recB* and *recC* mutant strains requires both the ATPase and nuclease activities of P2 OLD (Figure 6). We anticipate that these activities will also be required for the λ interference phenotype. Future genetic experiments examining the behavior of OLD ATPase and nuclease mutants both in this context as well as in other systems will help to unravel the biological functions of this diverse family of proteins.

## DATA AVAILABILITY

The atomic coordinates and structure factors of the Ts^FL^ structure are deposited in the Protein Databank with the accession number 6P74.

## Supplementary Material

gkaa059_Supplemental_File
