# Supplementary material for: The full-length structure of Thermus scotoductus OLD defines the ATP hydrolysis properties and catalytic mechanism of Class 1 OLD family nucleases
Source: Nucleic Acids Res. 2020 Feb 3;48(5):2762–76. doi: 10.1093/nar/gkaa059 (PMC7049728; doi:10.1093/nar/gkaa059)
Supplement: gkaa059_Supplemental_File [file gkaa059_supplemental_file.pdf]

Supplementary information for:

The full-length structure of *Thermus scotoductus* OLD defines the ATP hydrolysis properties and catalytic mechanism of Class 1 OLD family nucleases

Carl J. Schiltz[1], Myfanwy C. Adams[1], and Joshua S. Chappie[1,*]

[1] Department of Molecular Medicine, Cornell University, Ithaca, NY, 14853, USA

* To whom correspondence should be addressed. Tel: +1 (607) 253-3654; Fax: +1 (607) 253-3659; Email: chappie@cornell.edu

Supplementary Tables

Table S1. Inductively coupled plasma atomic emission spectroscopy (ICP-AES) analysis of Ts^{CTR} metal binding.

Table S2. X-ray data collection and refinement statistics.

Supplementary Figures

Figure S1. SEC-MALS analysis of Ts OLD constructs and metal-dependence of nuclease activity. Related to Figure 1.

Figure S2. Nuclease activity stimulation in response to calcium and magnesium. Related to Figure 1.

Figure S3. Modulation and directionality of Ts^{FL} nuclease activity. Related to Figure 1.

Figure S4. Crystal packing of Ts^{FL} and ion interactions. Related to Figures 2, 3, and 5.

Figure S5. Topology of OLD family nucleases. Related to Figures 2 and 5.

Figure S6. Sequence alignment of Class1 OLD family nucleases. Related to Figures 2, 3, and 5.

Figure S7. Conservation of Toprim residues at the ATPase domain interface. Related to Figure 2 and 5.

Figure S8. ATPase properties of Ts OLD. Related to Figure 3.

Figure S9. Ts OLD ATPase domain shares structural homology with genome maintenance proteins. Related to Figure 3.

Figure S10. Catalytic motifs of SMC/Rad50/RecN/RecF ABC ATPases. Related to Figures 3 and 4.

Figure S11. Organization of ATPase domain dimers. Related to Figure 4.

Figure S12. Representative spot assay of P2 OLD wildtype and mutants in $RecBC^{ts}$ *E. coli* at 30°C. Related to Figure 6.

Table S1. Inductively coupled plasma atomic emission spectroscopy (ICP-AES) analysis of Ts^{CTR} metal binding.

Milliequivalents of metal per protein molecule						
	Mg	Mn	Ca	Zn	Ni	Co
Ts^{CTR}	9.89	-0.01	69.72	0.90	4.07	0.00

Table S2. X-ray data collection and refinement statistics.

	Ts^{FL} Pt<br>PDB: 6P74
**Data collection**	
Space group	$I2_12_12_1$
Cell dimensions	
a, *b*, *c* (Å)	83.36, 101.74, 202.75
α, β, γ (°)	90, 90, 90
Resolution (Å)	101.38-2.12 (2.18-2.12)
R_{merge} (%)	9.6 (193.5)
R_{meas} (%)	9.7 (201.2)
$CC_{1/2}$ (%)	99.9 (23.6)
$I / \sigma I$	17.6 (0.5)
Completeness (%)	100 (99.8)
Redundancy	18.1 (18)
**Phasing**	
Initial F.O.M.	0.65
Number of sites	3
**Refinement**	
Resolution (Å)	101.38-2.20
No. reflections	44131 (3875)
R_{work} / R_{free} (%)	20.4/23.8
No. atoms	
Protein	4236
Ligand/ion	88
Water	66
B-factors	
Protein	88.4
Ligand/ion	189.9
Water	74.42
R.m.s deviations	
Bond lengths (Å)	0.009
Bond angles (°)	1.4
**Ramachandran statistics**	
Favored (%)	95.7
Allowed (%)	4.3
Outliers (%)	0

*Values in parentheses are for highest-resolution shell. Each dataset was derived from a single crystal.

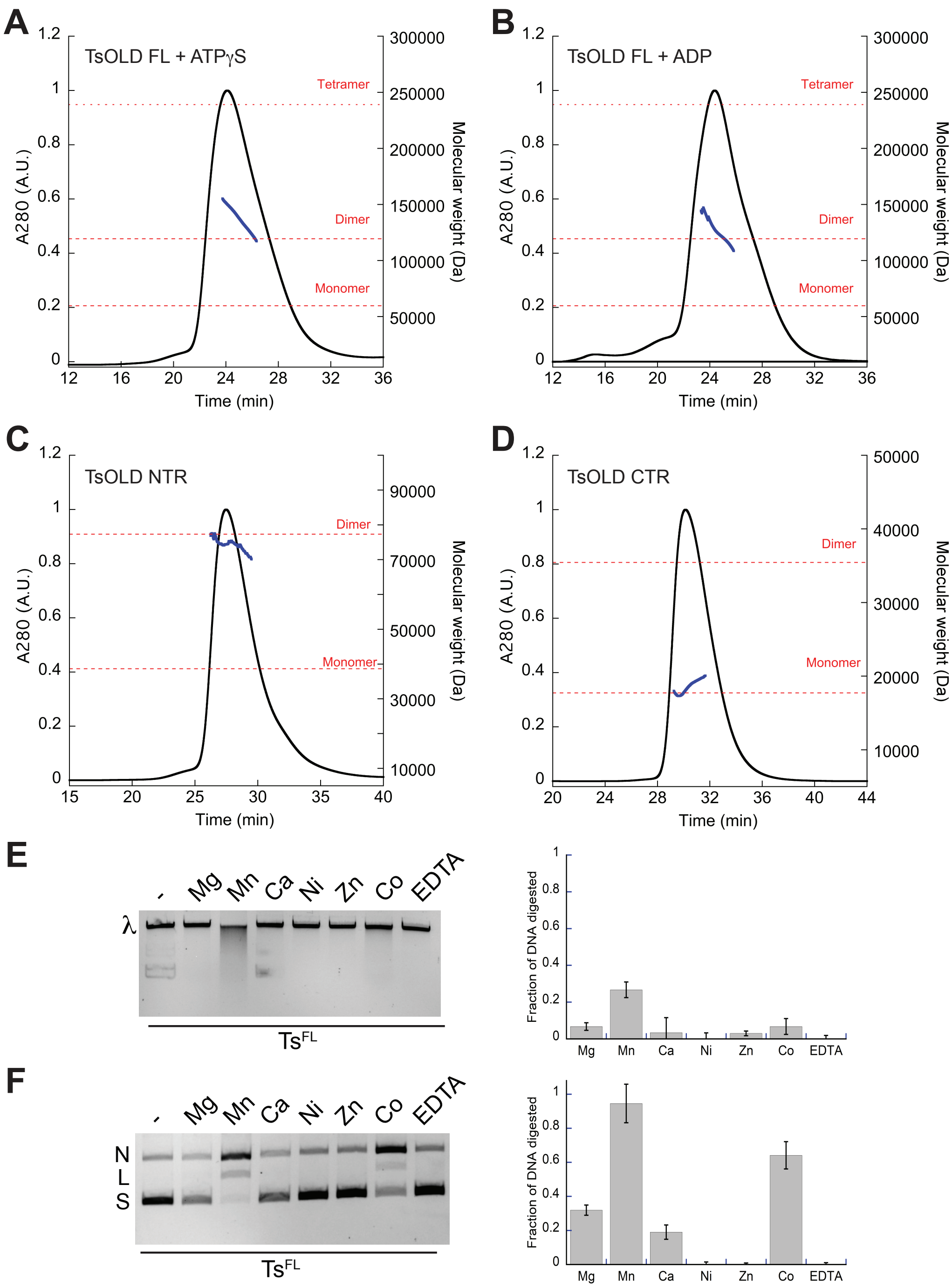

Figure S1. SEC-MALS analysis of Ts OLD constructs and metal-dependence of nuclease activity. A-D. SEC-MALS characterization of Ts OLD constructs in the presence (**A** and **B**) and absence (**C** and **D**) of different nucleotides. Construct abbreviations are as follows: FL, full-length (residues 1-525); NTR, N-terminal region (residues 1-369); CTR, C-terminal region (residues 370-525). UV trace (black) and calculated molecular weight based on light scattering (blue) are shown. Dashed red lines denote the molecular weight predicted for a monomer, dimer, and/or tetramer of each construct. **E,F.** Metal-dependent nuclease activity of Ts^{FL} over a five minute time course on linear λ (**E**) and supercoiled

pUC19 (**F**) DNA. N', 'L', and 'S' in (**F**) denote the positions of 'nicked', 'linearized', and 'supercoiled' DNA respectively. Representative gels and quantification of each experiment are shown. Graphs represent the average of three independent trials with error bars representing the standard error of the mean.

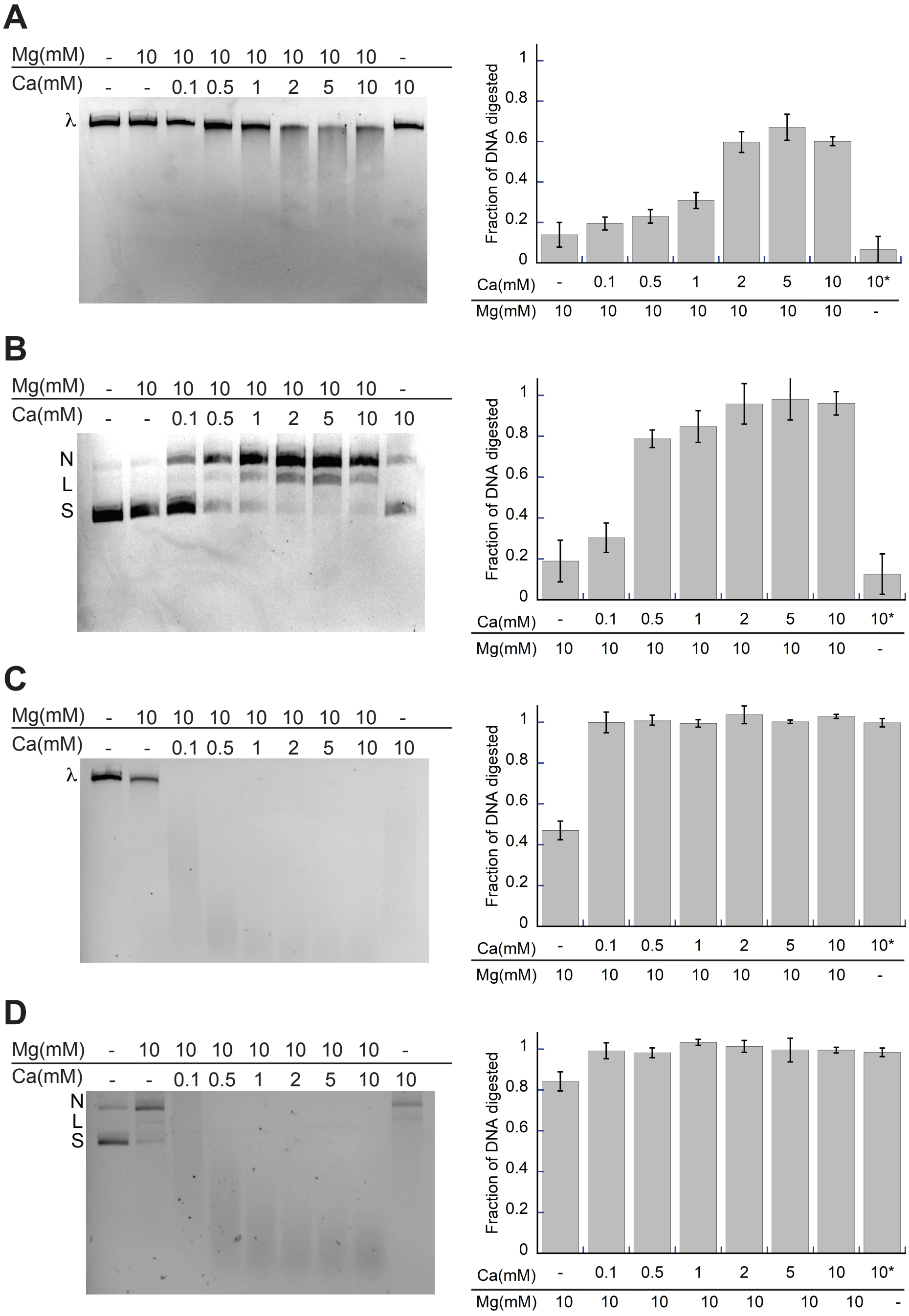

Figure S2. Nuclease activity stimulation in response to calcium and magnesium. A-D. Calcium and magnesium modulation of Ts^{FL} nuclease activity on linear λ (**A**) and supercoiled pUC19 (**B**) DNA. Samples were incubated at 65°C for 5 minutes. Ts^{FL} nuclease activity on linear λ (**C**) and supercoiled pUC19 (**D**) DNA after a 15 minute incubation at 65°C. Graphs represent the average of three independent trials with error bars representing the standard error of the mean.

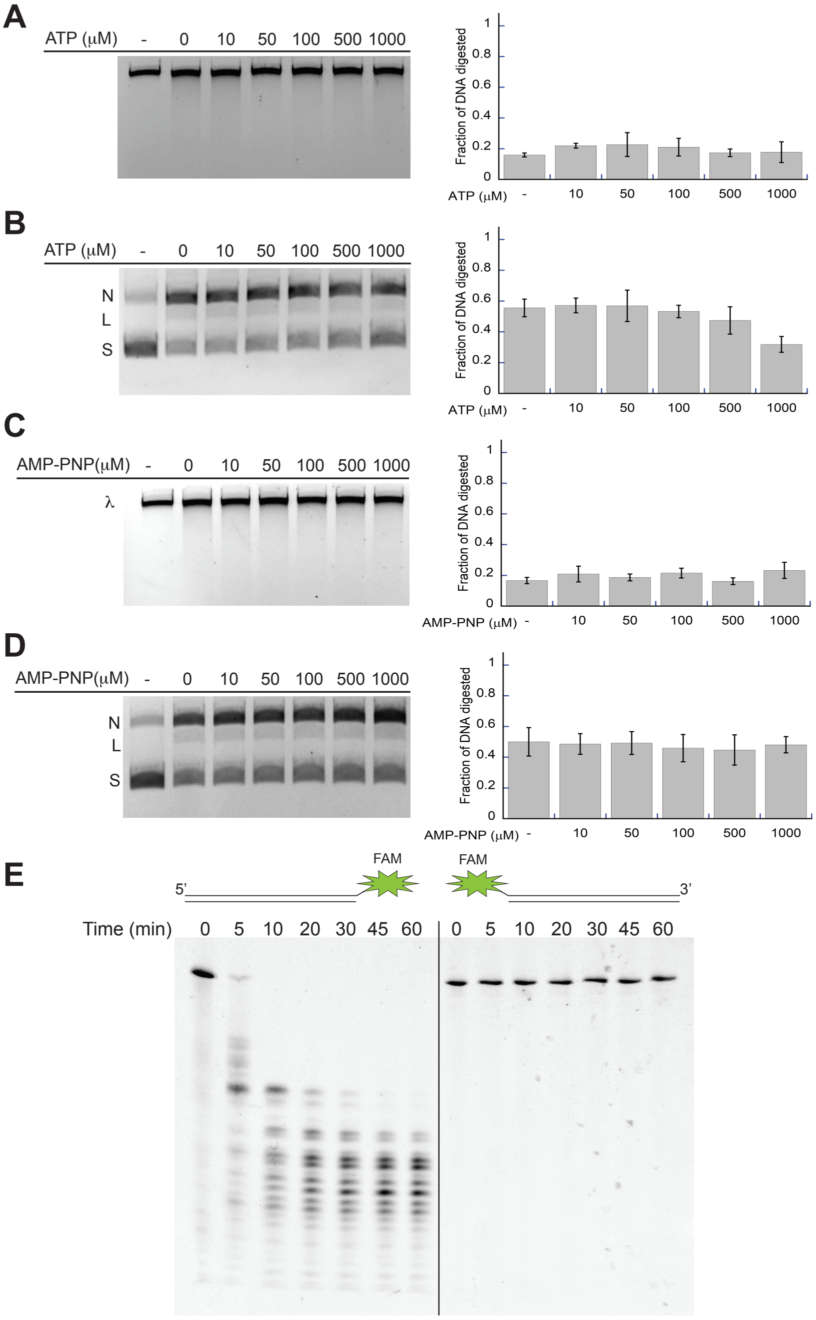

Figure S3. Modulation and directionality of Ts^{FL} nuclease activity. A-B. Representative gels and quantification of Ts^{FL} nuclease activity on linear λ (**A**) and supercoiled pUC19 (**B**) DNA in the presence of increasing amounts of ATP when incubated at 65°C for 5 minutes. **C-D.** Representative gels and quantification of Ts^{FL} nuclease activity on linear λ (**C**) and supercoiled pUC19 (**D**) DNA in the presence of increasing amounts of AMP-PNP. All graphs represent the average of three independent trials with error bars representing the standard error of the mean. **E.** Time course of TsFL digestion of a 70 bp DNA substrate with either the 3' (left) or 5' (right) end labeled with 6-carboxyfluorescein (6-FAM).

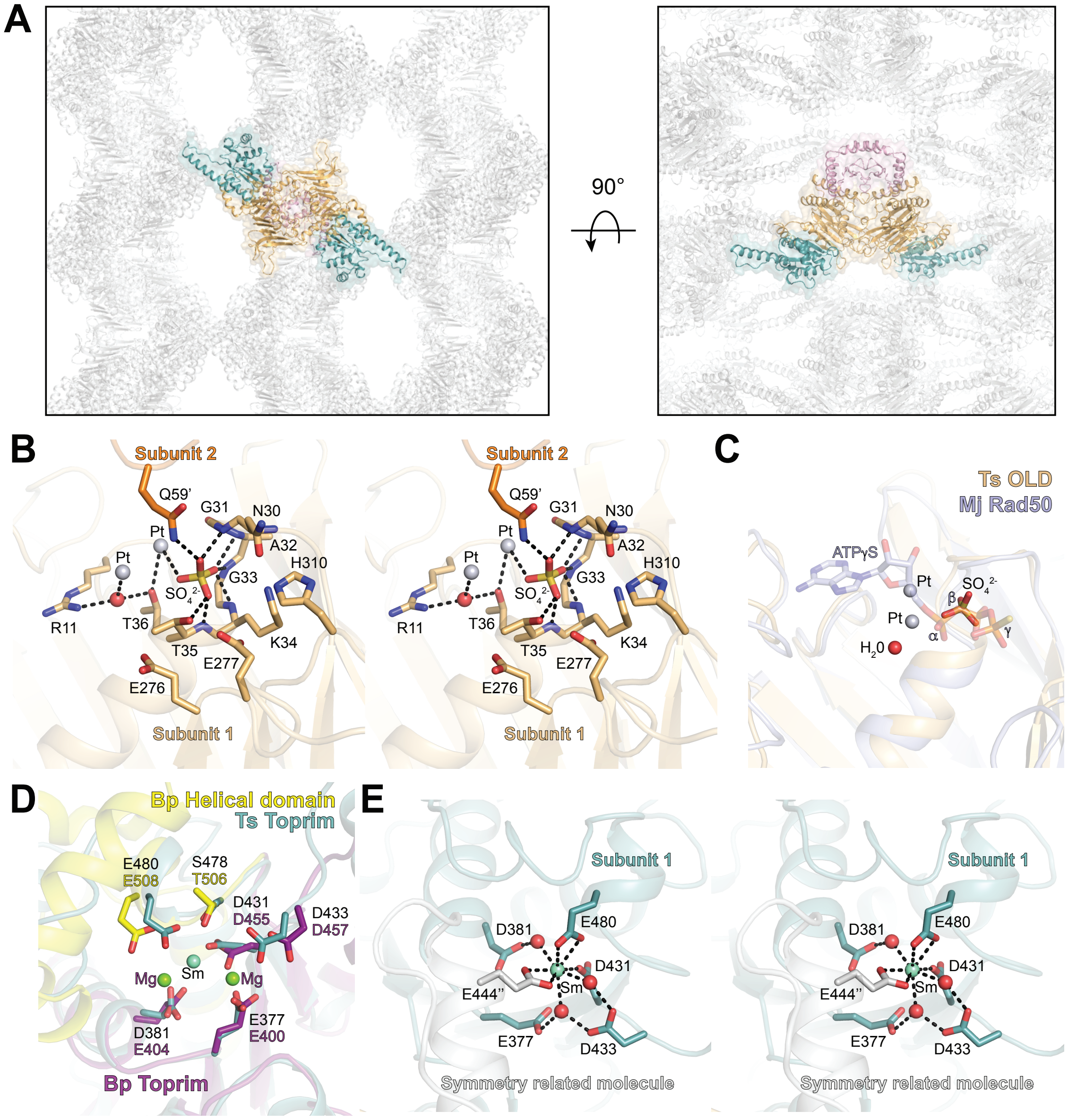

Figure S4. Crystal packing of Ts^{FL} and ion interactions. A. Individual molecules in the crystal lattices are colored gray with a single dimer highlighted and colored with ATPase domains in light orange, dimerization domains in pink, and Toprim domains in teal. **B.** Stereo view of Ts OLD ATP binding pocket. Ts OLD monomers are colored orange with bound platinum ions (gray) and ordered water molecules (red) shown as spheres and sulfate ion (yellow) shown as sticks. Hydrogen bond contacts are depicted as black dashes. Interacting residues are labeled and shown as sticks. **C.** Superposition of Ts OLD (orange) and *Methanococcus jannashii* (Mj) Rad50 (light blue; PDB: 5DNY) P-loop segments. ATPγS bound in Mj Rad50 is shown. Ts OLD ligands and ions are colored as in (**B**). **D.** Superposition of Ts OLD (teal) and the *Burkholderia pseudomallei* (Bp) OLD CTR (PDB: 6NK8). Bp OLD Toprim and helical domains are colored purple and yellow respectively. Active site residues critical

for metal binding are labeled in each structure. Relative positions of the bound metals are shown: samarium in Ts OLD, light green sphere; magnesium ions in Bp OLD, green spheres. **E.** Stereo view of Ts OLD Toprim interactions occurring between symmetry-related molecules (teal and white) in the crystal lattice. Light green sphere denotes bound samarium ion (Sm) that mediates crystal contacts. Side chains and water molecules (red spheres) that act as coordinating ligands are shown with black dashes indicating hydrogen bonds.

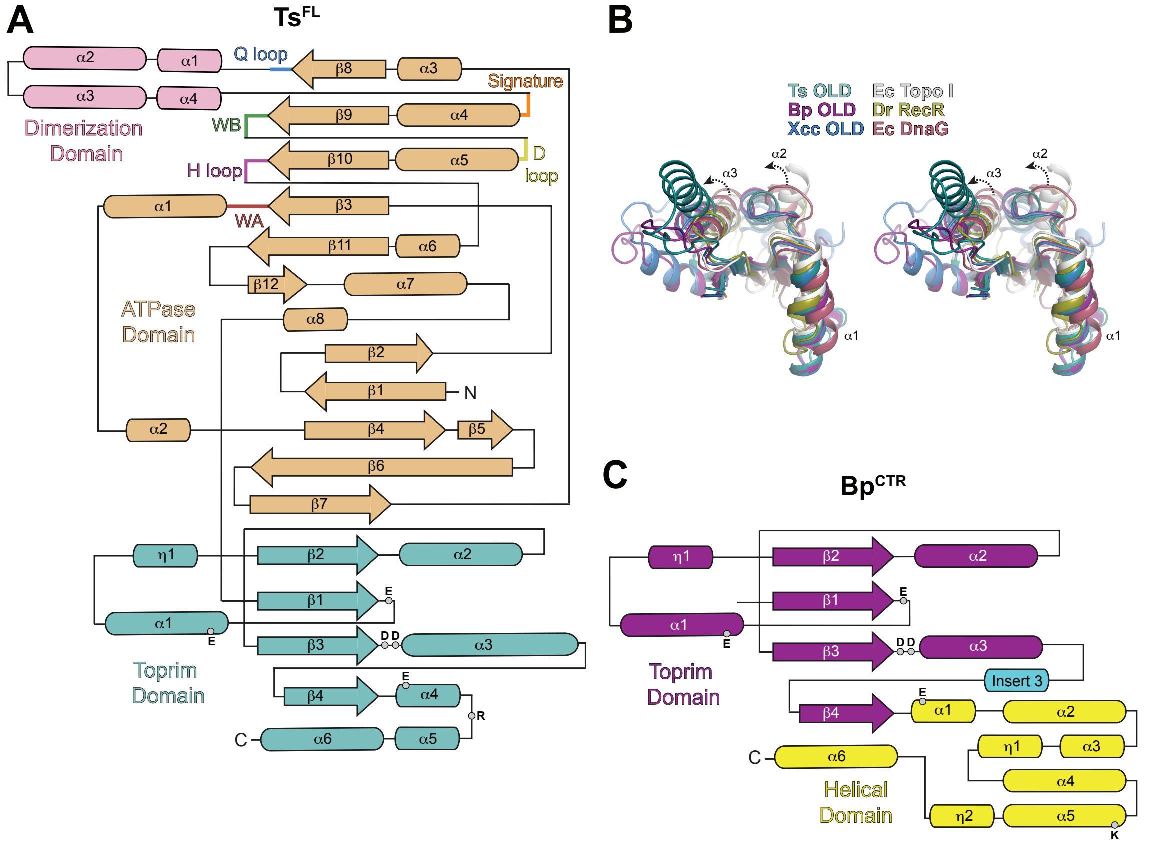

Figure S5. Topology of OLD family nucleases. A. Topology diagram of Ts^{FL} subunit. Coloring as follows: ABC ATPase domain, light orange; dimerization domain, pink; Toprim domain; teal; Ploop/Walker A (WA), red; Q loop, blue; Signature sequence, orange; Walker B (WB), green; D loop, yellow; H loop, purple. Location of Toprim catalytic residues responsible for nuclease cleavage are noted. **B.** Stereo view of structurally conserved α2 and α3 helical shifts (dashed arrows) in OLD family Toprim cores relative to the Toprim central β-sheet. Toprim cores for the following are shown for comparison: Ts OLD, teal; *Burkholderia pseudomallei* (Bp) OLD (PDB: 6NK8), dark purple; *Xanthomonas campestris* pv. *campestris* (Xcc) OLD (PDB: 6NJW), blue; *Escherichia coli* (Ec) Topoisomerase I (PDB: 1MW9), gray; *Deinococcus radiodurans* (Dr) RecR (PDB: 1VVD), olive; *Escherichia coli* DnaG (PDB: 3B39), raspberry. **C.** Topology diagram of Bp^{CTR}. Toprim and helical domains are colored dark purple and yellow respectively. Location of catalytic residues responsible for nuclease cleavage are noted. 'Insert 3' helix is colored cyan.

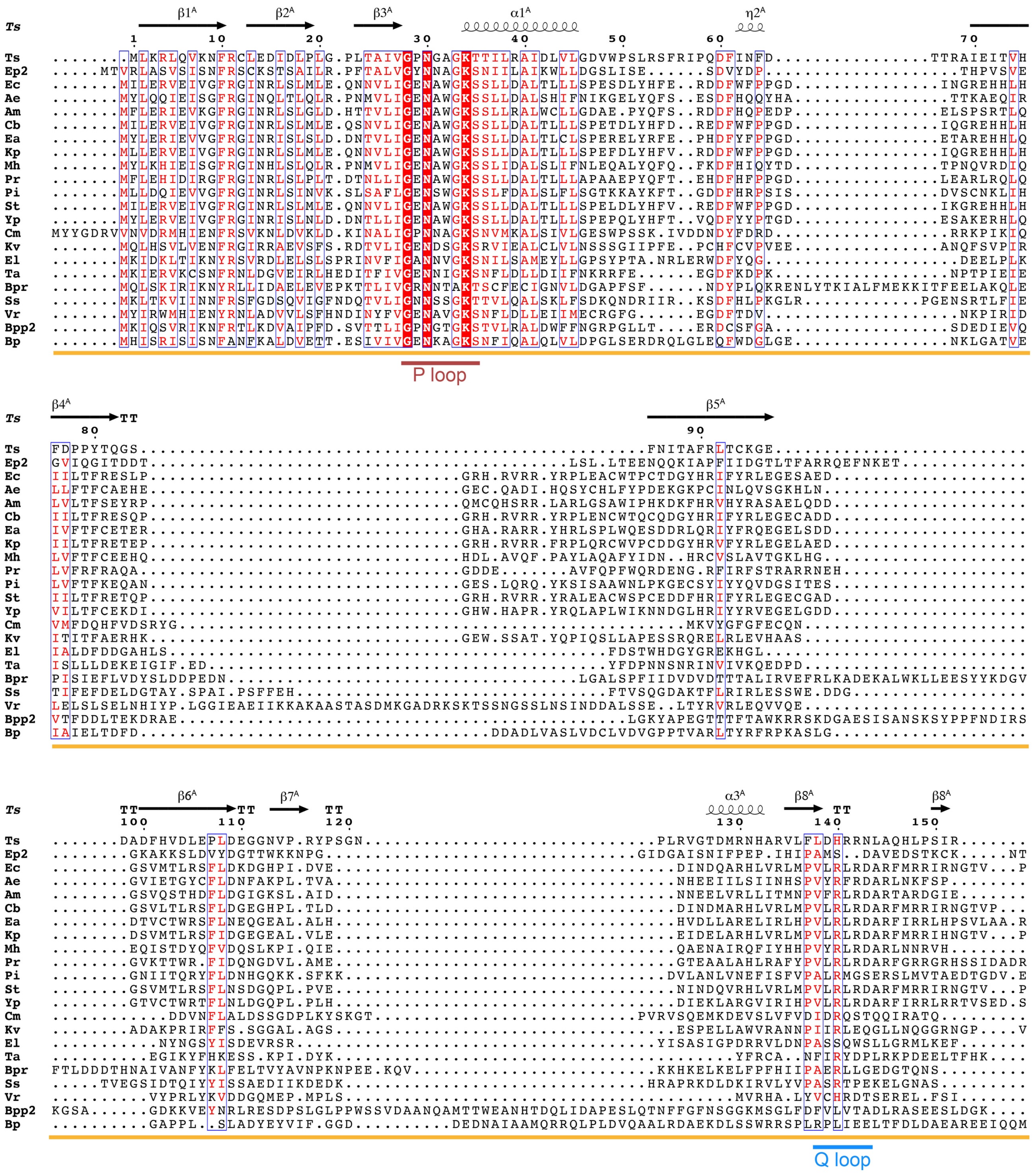

Figure S6. Sequence alignment of Class1 OLD family nucleases. Sequence alignment of representative Class 1 OLD family nucleases with secondary structure of Ts^{FL} mapped above. Orange, pink, and teal bars beneath the sequences denote the boundaries of the Ts ABC ATPase, dimerization, and Toprim domains respectively. Positions of the ATPase domain catalytic motifs (P loop, Q loop, Signature sequence, Walker B, D Loop, and H loop) are labeled beneath the sequences and colored as in Supplementary Figures S5, S10, and S11. Stars denote positions of metal A and metal B coordinating residues and R487. Sequence shading indicates conservation: white text on red background, 100% conserved; boxed red text on white background, 70% conserved. Abbreviations are as follows with accompanying KEGG IDs: Ts, *Thermus scotoductus* (tsc:TSC_c04750)*;* Ep2,

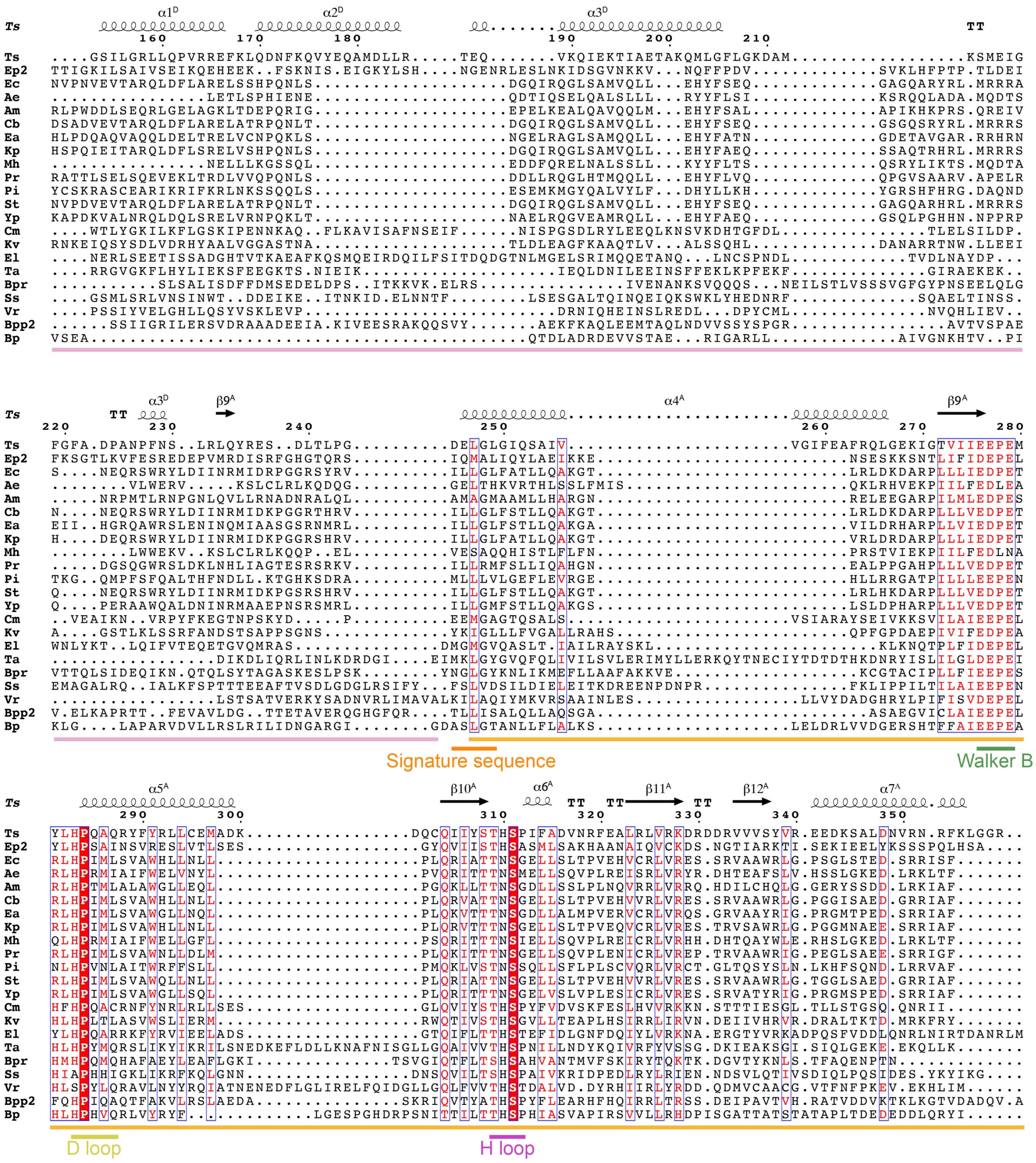

Escherichia coli phage P2 (vg:1261523); Ec, *Escherichia coli* (ecj:JW0860); Ae, *Actinobacillus equuli* (aeu:ACEE_06610)*;* Am, *Aeromonas media* (amed:B224_2448); Cb, *Citrobacter braakii* (*cbra:A6J81_00640);* Ea, *Edwardsiella anguillarum* (ete:ETEE_0205)*;* Kp, *Klebsiella pneumoniae* (kpp:A79E_3329); Mh, *Mannheimia haemolytica* (mhaq:WC39_10155); Pr, *Pantoea rwandensis* (kln:LH22_15680); Pi, *Psychromonas ingrahamii* (pin:Ping_0201); St, *Salmonella enterica* (sty:STY0935); Yp, *Yersinia pestis* Angola (ypg:YpAngola_A1594); Cm, *Candidatus Micrarchaeota* (marh:Mia14_0874); Kv, *Koribacter versatilis* (aba:Acid345_4083); El, *Eggerthella lenta* (ele:Elen_0801); Ta, *Thermocrinis albus* (tal:Thal_0492); Bpr, Butyrate-producing bacterium SS3/4

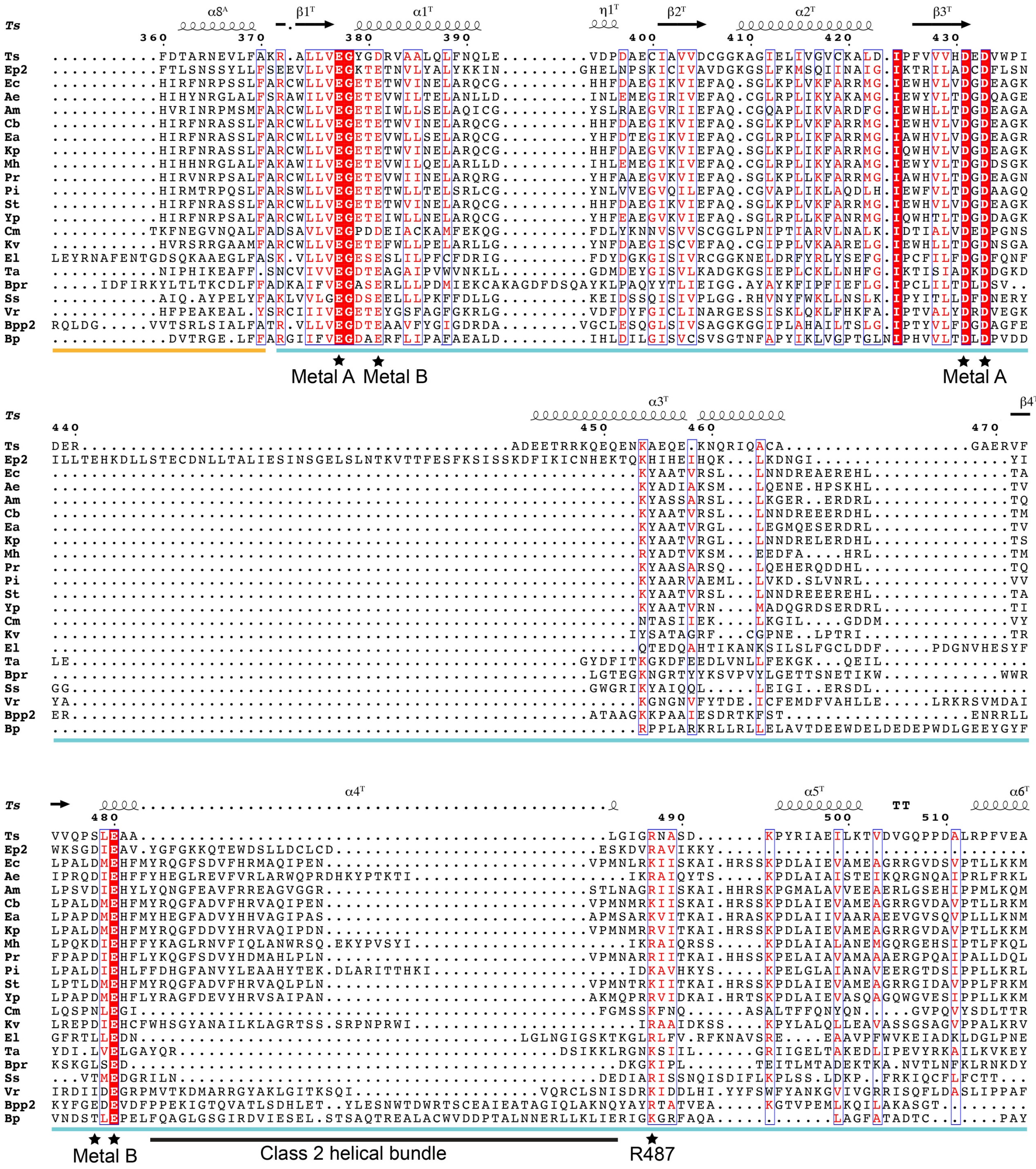

(bprs:CK3_32330); Ss, *Stroptococcus suis* (ssv:SSU98_0942); Vr, *Veillonella rodentium* (vrm:44547418_01362); Bpp2, *Burkholderia pseudomallei* phage P2 (vg:10323863). The Class 2 OLD homolog *Burkholderia pseudomallei* (Bp, bpd:BURPS668_A0038) is included for comparison.

```
Ts      ℓℓℓℓℓ
      520
Ts      IRQVTRPMEE.........................
Ep2     ........DEMEDFIKWI.................
Ec      FSRVLWLARGRAD......................
Ae      FIKVLQLIKEQ........................
Am      FARVVALARGQS.......................
Cb      FSRVLWLARGRAD......................
Ea      FSRVLWLARGKAD......................
Kp      FSRVLWLARGRAD......................
Mh      FADVLALINKV........................
Pr      FARVMWLARGRAD......................
Pi      FSRLVGLARSQSG......................
St      FSRVVWLARGRAD......................
Yp      FSRVVWLARGRADE.....................
Cm      LSP................................
Kv      IESCVRMARRTNEQSAPDVTQPALKDNLLHMEARK
El      ARSVLTCKCEPLAWDDDYIPF..............
Ta      VESQTGGSAGE........................
Bpr     AATPWKKLSGMSIVRIMNWRTL.............
Ss      ...................................
Vr      IAVVERAKTLSLGLA....................
Bpp2    ...................................
Bp      IRNALEYIRDAVA......................
```

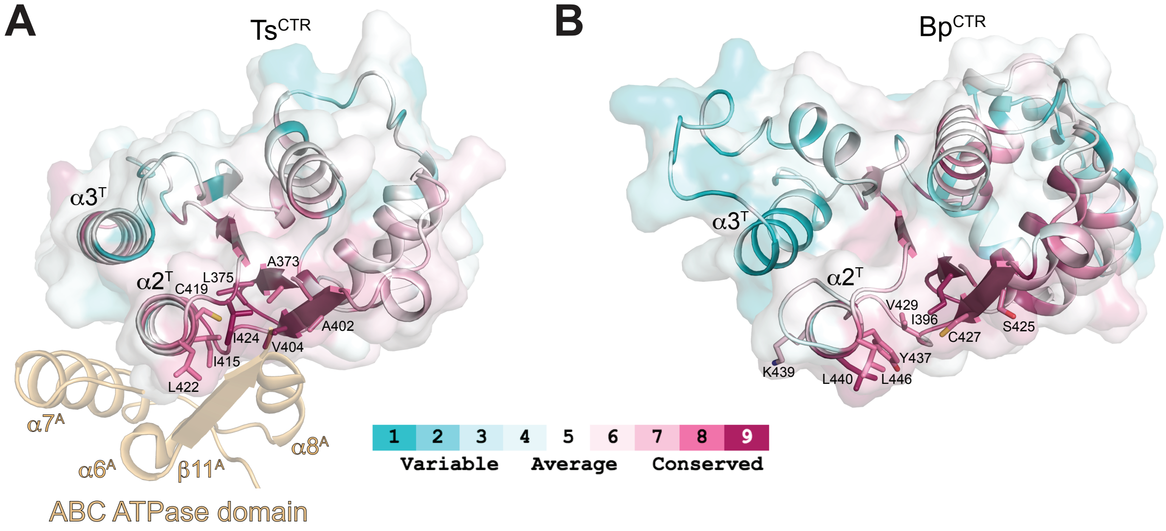

Figure S7. Conservation of Toprim residues at the ATPase domain interface. A. Highly conserved Toprim residues outside the nuclease active site map to the interface between the Ts ATPase domain (light orange) and the Ts^{CTR}. Ts^{CTR} coloring (see legend below) reflects sequence conservation and was generated using the ConSurf server (14) and alignment in Supplementary Figure S7. **B.** Highly conserved residues in **A** are spatially conserved in Bp^{CTR} (PDB: 6NK8; adapted from Schiltz et al., accompanying manuscript).

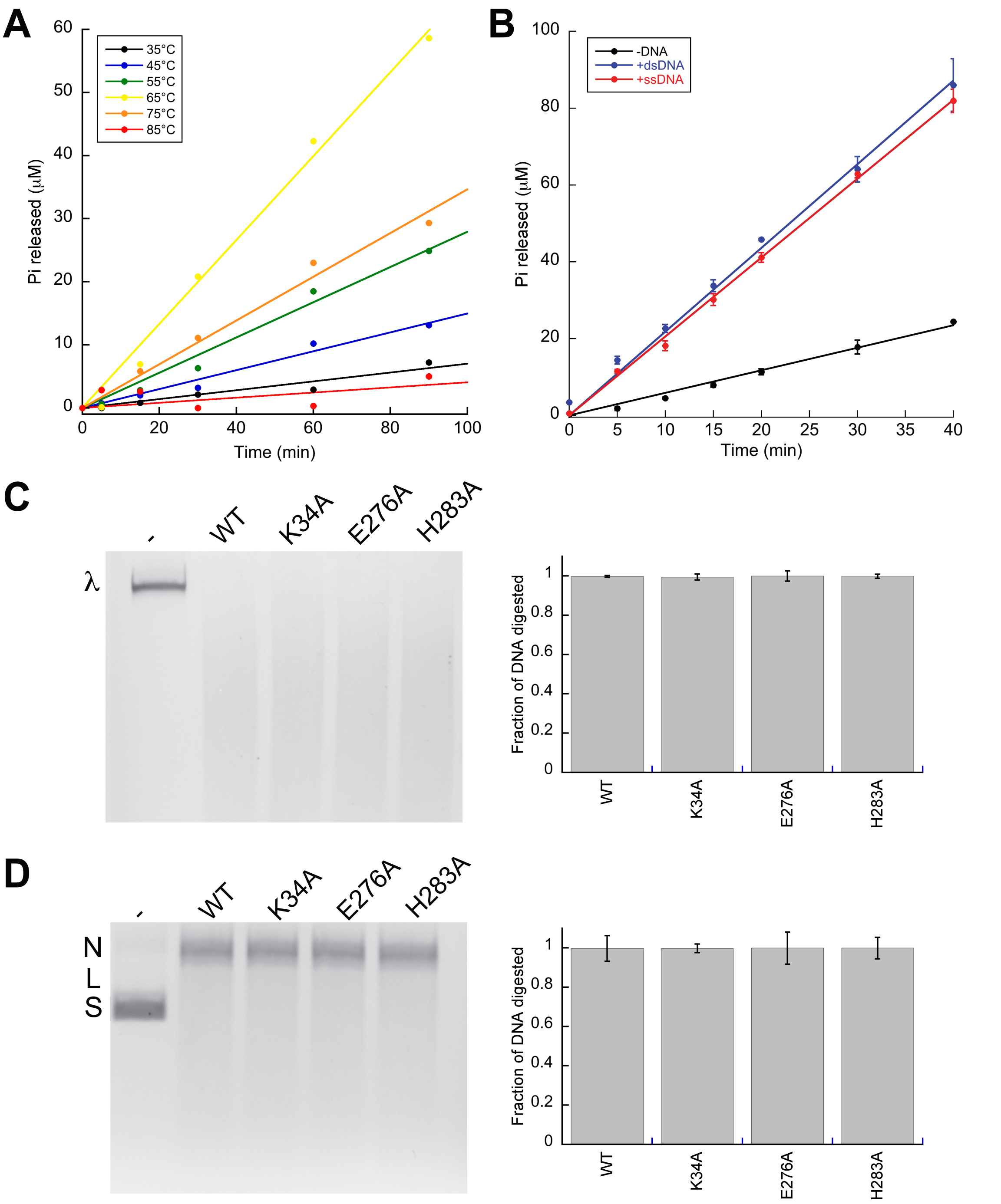

Figure S8. ATPase properties of Ts OLD. A. Temperature dependence of Ts^{FL} ATPase activity. **B.** DNA stimulates Ts^{FL} ATPase activity. Reactions were carried out with 0.5 mM ATP at 65°C in the absence (-DNA, black) or presence of a 70-mer single-stranded (ssDNA, red) or double-stranded (dsDNA, blue) DNA substrate added at of 8 μM. **C-D.** Nuclease activity of Ts^{FL} ATPase mutants on linear λ (**C**) and supercoiled pUC19 (**D**) DNA. N', 'L', and 'S' denote the positions of 'nicked', 'linearized', and 'supercoiled' DNA respectively. All graphs represent the average of three independent trials with error bars representing the standard error of the mean.

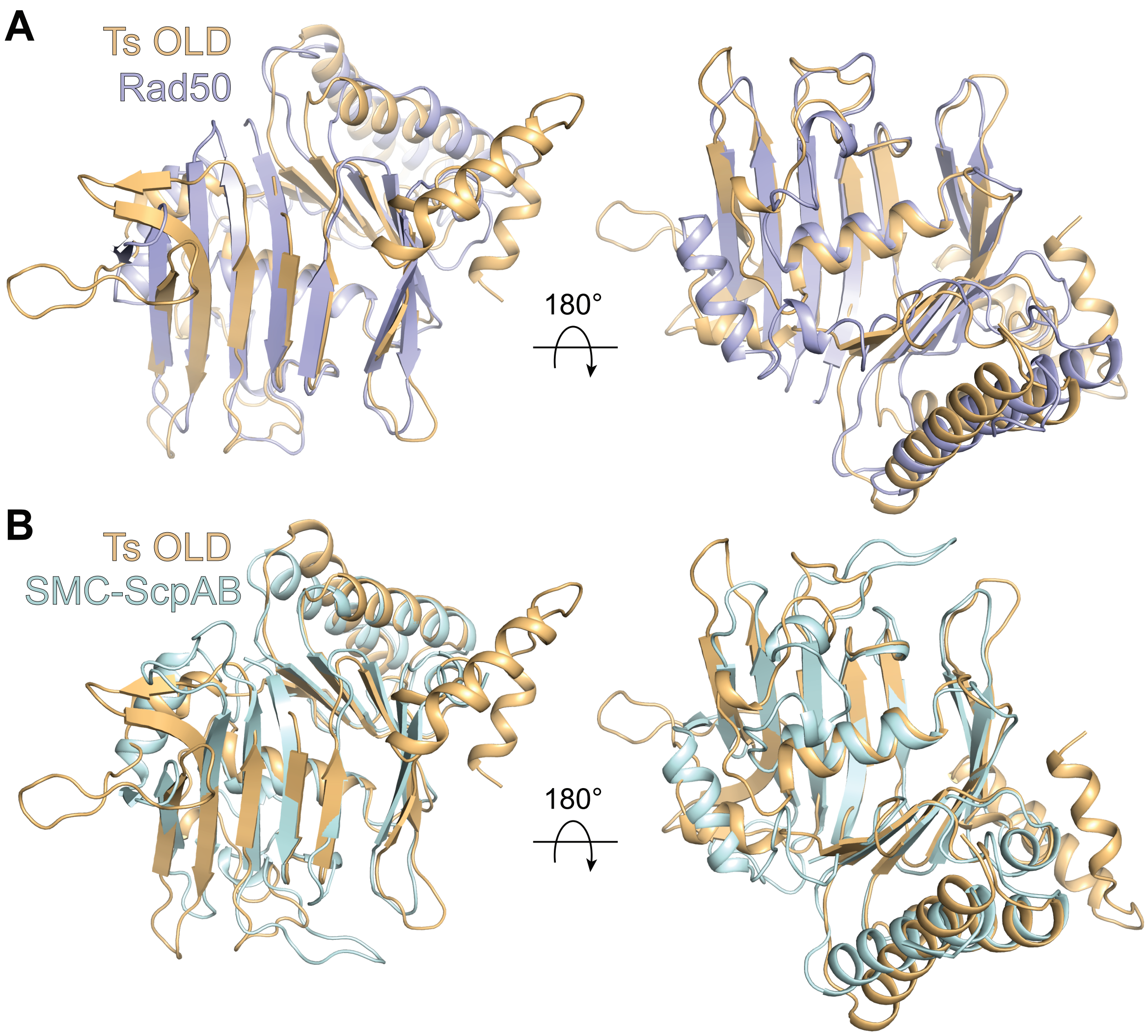

Figure S9. Ts OLD ATPase domain shares structural homology with genome maintenance proteins. A-B. Structural superposition of the Ts OLD ATPase domain the ATPase domain (light orange) from *Methanococcus jannashii* Rad50 (**A**, light blue; PDB: 5DNY) and SMC-ScpAB complex from *Pyrococcus furiosus* (**B**, pale cyan, PDB: 4I99).

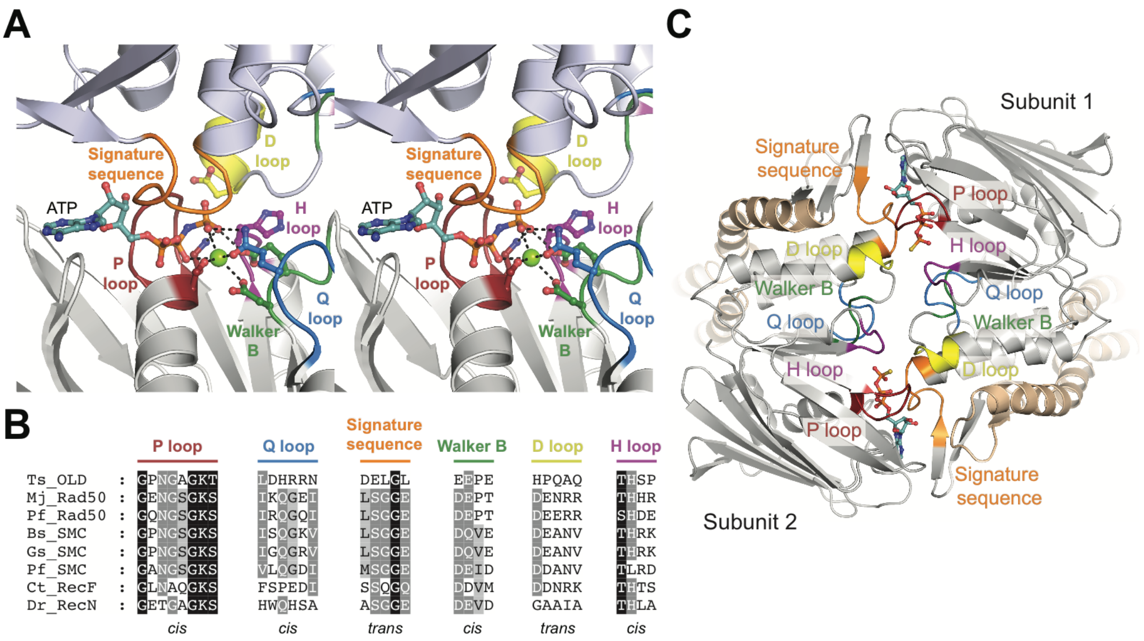

Figure S10. Catalytic motifs of SMC/Rad50/RecN/RecF ABC ATPases. A. Stereo view of *Pyrococcus furiosus* Rad50 (PDB: 3QKU) active site. Subunits are colored white and blue white respectively, with sequence motifs contributing to ATP binding and hydrolysis colored as follows: P loop, red; Q loop, blue; ABC signature sequence, orange; Walker B, green; D loop, yellow; H loop, purple. ATP substrate is colored light teal while bound magnesium cofactor is depicted as a green sphere. Catalytic side chains from each motif are shown as sticks with hydrogen bonding interactions illustrated with black dashes. **B.** Sequences of ABC ATPase motifs from structurally characterized SMC/Rad50/RecN/RecF family members. Shading denotes conservation between these representatives. Contribution of each motif, either *in cis* or *in trans*, in indicated below. Nomenclature and associated PDB codes are as follows: *Thermus scotoductus* (Ts) OLD; *Methanocaldococcus jannaschii* (Mj) Rad50 (PDB: 5DNY); *Pyrococcus furiosus* (Pf) Rad50 (PDB: 3QKU); Bacillus subtilis (Bs) SMC (PDB: 5XG3); *Geobacillus stearothermophilus* (Gs) SMC (PDB: 5H68); *Pyrococcus furiosus* SMC (PDB: 4I99); *Caldanaerobacter subterraneus subsp. tengcongensis* (Ct) RecF (PDB: 5z68); *Deinococcus radiodurans* (Dr) RecN (PDB: 4ABY). **C.** Bottom view Mj Rad50 NBD dimer (PDB: 5DNY) illustrating the interaction and proximity of the catalytic motifs from each subunit in the canonical head-to-head arrangement. ATPase core and helical coils colored gray and wheat respectively. ABC motifs are colored in as in A and labeled.

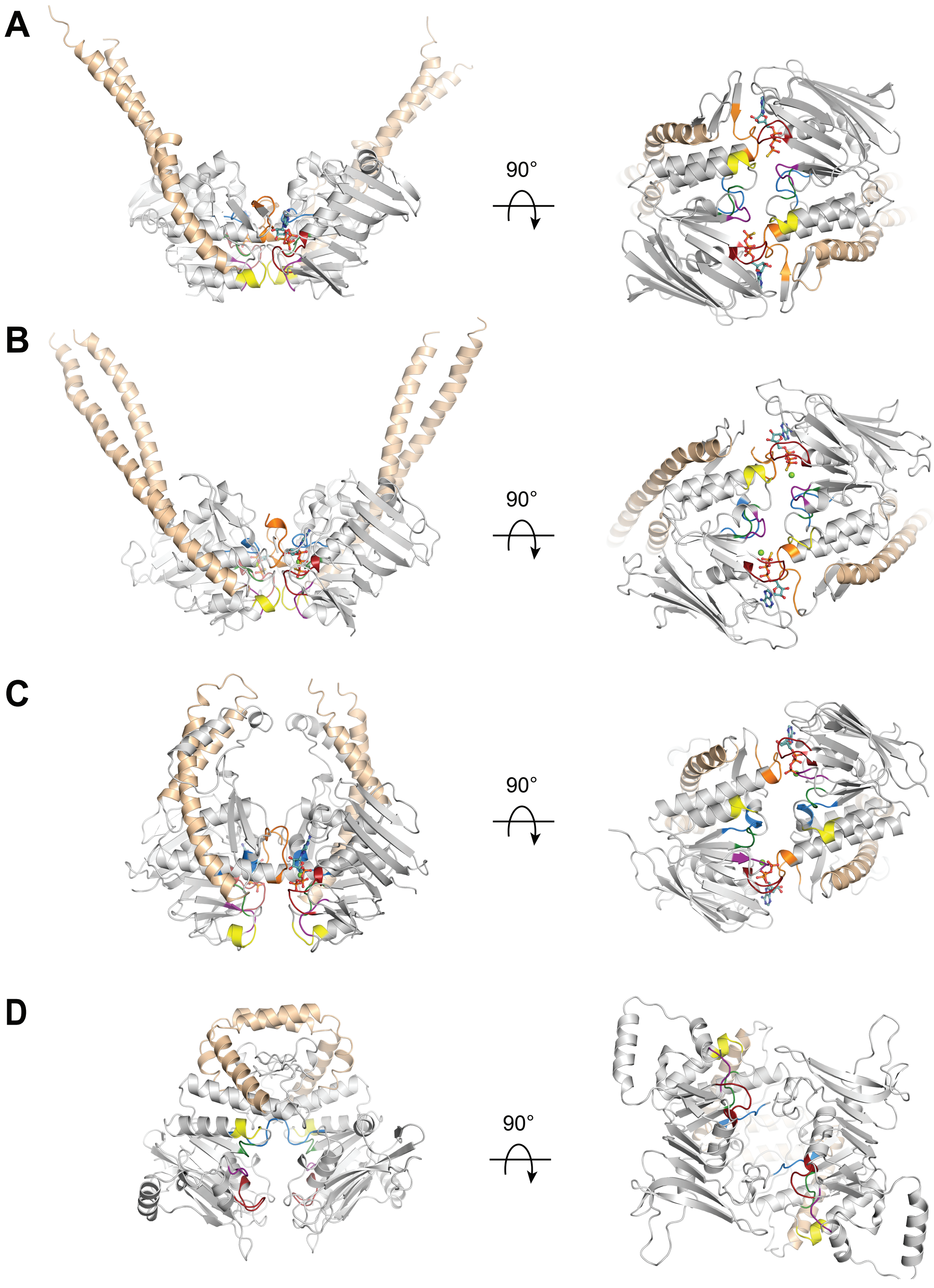

Figure S11. Organization of ATPase domain dimers. A-D. Organization of ATPase domains in the dimers of *Methanocaldococcus jannaschii* Rad50 (PDB: 5DNY) (**A**), *Bacillus subtilis* SMC (PDB: 5XG3) (**B**), *Caldanaerobacter subterraneus subsp. tengcongensis* RecF (PDB: 5z68) (**C**), and *Thermus scotoductus* (Ts) OLD (**D**). ABC ATP domain core is colored gray with the extended coils colored wheat to illustrate the relative orientation of each subunit. Motifs involved in nucleotide binding and hydrolysis

colored as follows: P loop, red; Q loop, blue; ABC signature sequence, orange; Walker B, green; D loop, yellow; H loop, purple. Bound nucleotides are colored light teal while bound magnesium ions are depicted as green spheres. Side (left) and bottom (right) views of each dimer are shown.

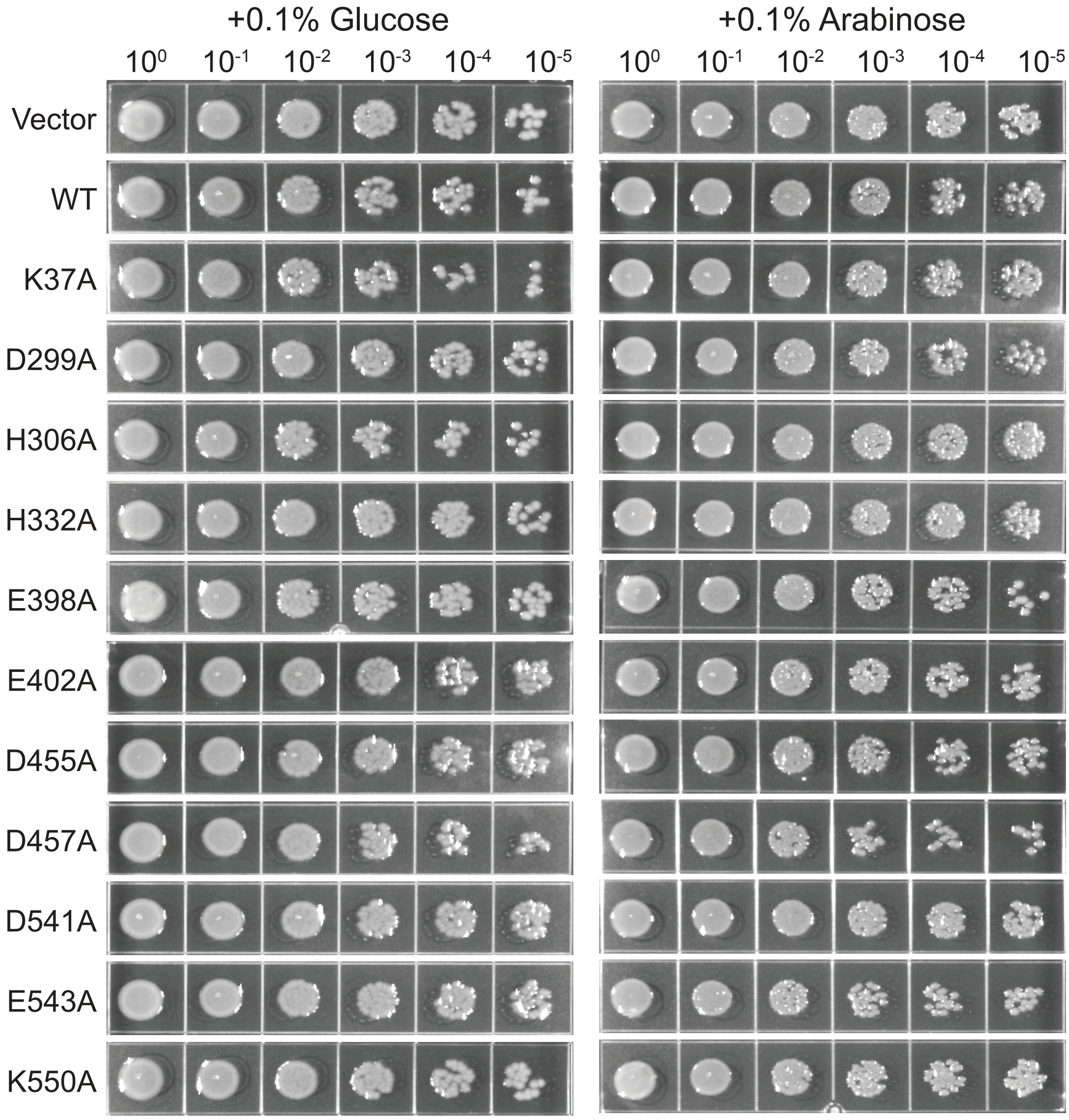

Figure S12. Representative spot assay of P2 OLD wildtype and mutants in RecBC^{ts} *E. coli* at 30°C. RecBC^{ts} *E. coli* transformed with arabinose-inducible P2 OLD wildtype as well as P2 OLD carrying mutations in the ATPase and nuclease domains were grown overnight at 30°C, the temperature permissive for RecBCts function. There is no noticeable loss of cell viability with P2 OLD expression induced (+0.1% arabinose) compared to P2 OLD expression repressed (+0.1% glucose).
